# Successful strategies that address gender-related barriers and promote bodily autonomy within efforts to scale up and sustain postpregnancy contraception: a scoping review

**DOI:** 10.1136/bmjgh-2024-016638

**Published:** 2025-02-16

**Authors:** Arachu Castro, Rita Kabra, Anna Coates, James Kiarie

**Affiliations:** 1 International Health and Sustainable Development, Tulane University School of Public Health and Tropical Medicine, New Orleans, Louisiana, USA; 2 World Health Organization, Geneva, Switzerland

**Keywords:** Global Health, Health policies and all other topics, Health services research, Public Health

## Abstract

**Introduction:**

Acknowledging the integral role of bodily autonomy in advancing gender equality, our study aimed to assess the extent to which strategies used in postpartum and postabortion contraception have effectively equipped women, girls and gender-diverse individuals with the tools, knowledge and resources required to make autonomous decisions that align with their diverse life experiences.

**Methods:**

We conducted a scoping review using the databases PubMed, EBSCOhost, EMBASE and SciSpace. We included implementation, evaluation and experimental studies published in any language between 2013 and 2023 and excluded studies not meeting these criteria. We used a WHO scale to determine the level of gender responsiveness.

**Results:**

We found 30 implementation, evaluation and experimental studies published in any language between 2013 and 2023. We categorised the strategies following the WHO scale as gender-transformative (4 studies), gender-specific (24 studies) and gender-sensitive (2 studies). None of the studies reported strategies hindering reproductive health and rights. All strategies involved women and girls, and none explicitly targeted gender-diverse people capable of childbearing.

**Conclusions:**

This study highlights the importance of integrating gender-transformative activities into postpregnancy contraceptive strategies and underscores the necessity of understanding and addressing local gender norms and the broader health system context to promote bodily autonomy effectively. The findings suggest that success should not be solely measured by contraceptive uptake but also by how well interventions address gender-related barriers.

**WHAT IS ALREADY KNOWN ON THIS TOPIC**

- Previous scoping and systematic reviews have explored strategies to increase postpregnancy contraception uptake globally. However, none have specifically focused on strategies that promote bodily autonomy while addressing gender-related barriers. Our study addresses this gap by providing a comprehensive understanding of such strategies and their impact on scaling up and sustaining postpregnancy contraception.

**WHAT THIS STUDY ADDS**

- This study provides new insights by being the first scoping review to focus on strategies promoting bodily autonomy in addressing gender-related barriers to scaling up and sustaining postpregnancy contraception. The gender-transformative strategies reported in the studies shared a commitment to empowering women with the autonomy to make informed decisions about postpregnancy contraception through (a) delivering personalised counselling that respected each woman’s reproductive goals and ensured privacy during these discussions; (b) integrating the cultural and familial context of women’s health decisions and (c) promoting a rights-based approach that prioritised informed consent and defended women’s reproductive rights.

**HOW THIS STUDY MIGHT AFFECT RESEARCH, PRACTICE OR POLICY**

- This study highlights the importance of integrating gender-transformative activities into postpregnancy contraceptive strategies. It underscores the necessity of understanding and addressing local gender norms and the broader health system context to effectively promote bodily autonomy. The findings suggest that success should not be solely measured by contraceptive uptake but also by how well interventions address gender-related barriers. Future research should focus on developing and validating indicators that evaluate these barriers and promote bodily autonomy, ensuring comprehensive strategies that truly empower women, girls and gender-diverse individuals with the means, abilities and assets to make informed choices that resonate with the broader spectrum of their lives.

## Introduction

 Within a framework of sexual and reproductive health (SRH) and rights, the WHO aims to fortify the gender-responsive approaches in the escalation and preservation of postpregnancy contraceptive services that contribute to a fulfilling fertility desire.^1^ This endeavour stems from recognising that reproductive and sexual freedom is foundational to gender equality and that ‘any individual has the necessary agency to determine their reproductive decisions and sexuality, regardless of gendered norms and gender-based discrimination’.^2^ Such freedom paves the way for women, girls and those of diverse gender identities to gain comprehensive control over their bodies and life trajectories, irrespective of prevailing restrictive gender perceptions related to sexuality, reproduction or any gender-related biases. Consequently, the availability of gender-responsive contraception, which goes beyond merely aiming to improve uptake or access to contraceptives but more ambitiously aims to promote personal bodily sovereignty, becomes indispensable in our quest for gender equality.

Health systems are often structured around service-centred approaches characterised by systemic shortcomings that perpetuate gender biases and undermine bodily autonomy. To address these issues, some authors have advocated for human rights-based reforms that prioritise bodily autonomy, tackle structural inequities and reorient health services to empower individuals and promote equity.^3^ Studies from around the world have established significant associations between indicators that reflect bodily autonomy in exercising SRH and rights and factors associated with women’s empowerment—such as higher education attainment, wealth quintile and paid work—as observed in Bangladesh,^4^ Mauritania,^5^ Nepal,^6^ Nigeria,^7^ Vietnam^8^ and the Latin American region.^9^ These findings underscore the intricate relationship between health systems and social structures, illustrating how public health strategies aimed at improving SRH have the potential to foster gender equality by enabling greater access to education and autonomy in decision-making.^10^

Accordingly, our study aims consist of evaluating if and how the structure and provision of postpartum and postabortion contraceptive services empower women, girls and gender-diverse individuals with the means, abilities and assets to make informed choices that resonate with the broader spectrum of their lives and challenges harmful gender norms, which may restrict reproductive and life choices. We focus on discerning how such interventions can effectively and intentionally foster bodily autonomy as a primary goal. The evidence generated through this review will advance the knowledge of strategies that successfully address gender-related barriers to scaling up and sustaining postpartum and postabortion contraception in ways that promote the exercise of bodily autonomy.

## Methodology

This scoping review follows the Preferred Reporting Items for Systematic Reviews and Meta-Analyses (PRISMA) Statement,^11^ the PRISMA Extension for Scoping Reviews guidelines^12^ and an adaptation of the Arksey and O’Malley^13^ framework for conducting scoping reviews.^14^ The scoping review and its protocol are registered in the OSF repository.^15^ The guidelines include several stages:

### Identifying the research question

We searched for evidence of addressing gender-related barriers and promoting bodily autonomy in successful strategies to scale up and sustain postpartum and postabortion contraception. In the absence of other measures in most impact evaluations, we defined success according to the metrics defined for most interventions, that is, increased contraceptive uptake postpregnancy, therefore, increasing access to SRH services.

### Identifying relevant studies

We searched PubMed, EBSCOhost (Academic Search Complete) and EMBASE online databases. Search terms “in any field” included: ((postpartum contraception) OR (postpartum family planning) OR (post abortion contraception) OR (post abortion family planning) OR (prenatal contraception) OR (prenatal family planning) OR (antenatal contraception) OR (antenatal family planning)) AND ((gender barrier) OR (gender issues) OR (cultural issues)). We also searched the database SciSpace, asking: “What are successful strategies for expanding postpartum or post-abortion contraception?” We included implementation, evaluation and experimental studies—studies that examined the delivery, effectiveness and impact of public health interventions—published in any language between 2013 and 2023. We excluded papers that did not meet these criteria. One researcher (ACa) conducted all the searches and the following steps in October 2023.

#### Study selection

We refined the research strategy in a set of steps. First, we imported all the references to EndNote V.20 and deleted duplicates. Second, we imported the remaining references to Covidence and conducted title and abstract screening. We removed publications that were either irrelevant, not found, abstract only, editorials or letters. Third, we imported the full version of the remaining references plus additional references found through citation search to SciSpace and asked: “Does the paper identify successes to postpartum or post-abortion contraception?” If positive, we refined the search by asking two follow-up questions tested multiple times to capture as many eligible papers as possible: “Were any successes identified?” and “Was the intervention successful?” We excluded the papers that yielded a negative response. We selected the remaining papers for full review and classified them for full review according to the type of studies. We retained implementation, evaluation and experimental studies and discarded reviews and observational studies. We assessed as eligible the publications that have successfully addressed barriers to the broad adoption and ongoing use of postpregnancy contraceptive methods, emphasising the reinforcement of bodily autonomy. One researcher (ACa) collected and reviewed the data.

### Charting the data

For each publication, we extracted information on the country where the research was conducted, the population studied and the methodological design. We charted the data according to four themes:

1. The level of gender responsiveness. To determine the level of each study, we used WHO’s Gender Assessment Tool and Gender Responsive Assessment Scale^16^ and additional considerations specific to SRH,^2^ shown in table 1.
2. Characteristics of gender-responsive strategies that reported success in expanding postpregnancy contraception, especially those for which impact evaluations have been conducted.
3. Key components of gender-transformative strategies that can be adapted to different contexts and/or scaled up.
4. Factors associated with using digital tools to promote postpregnancy contraceptive use.

**Table 1:** Levels of gender responsiveness according to the WHO’s Gender Responsive Assessment Scale and gender responsiveness of postpregnancy contraception services

Gender responsive assessment scale				Gender responsiveness of post-pregnancy contraception programmes
Level 1	Gender-unequal	Perpetuates gender inequality by reinforcing unbalanced norms, roles and relations and often leads to one sex enjoying more rights or opportunities than the other.	Hinder the achievement of gender equality and health equity.	Hinder the achievement of sexual and reproductive health and rights.
Level 2	Gender-blind	Ignores gender norms, roles and relations and very often reinforces gender-based discrimination. By ignoring differences in opportunities and resource allocation for women and men, such policies are often assumed to be ‘fair’ as they claim to treat everyone the same.		
Level 3	Gender-sensitive	Indicates gender awareness, although no remedial action is developed.	Represents a starting point for gender programming.	Designed to increase access to adequate postpregnancy contraception without addressing gender norms and relations. Results are only based on service delivery and specific health outcomes without considering autonomy or informed decision-making.
Level 4	Gender-specific	Considers women’s and men’s specific needs and intentionally targets and benefits a specific group of women or men to achieve certain policy or programme goals or meet certain needs. Such policies often make it easier for women and men to fulfil duties that are ascribed to them based on their gender roles but do not address underlying causes of gender differences.	Represent characteristics of gender-responsive health policies and programmes.	Designed to increase access to appropriate sexual and reproductive health resources through the provision of postpregnancy contraception. They address gender norms and relations and/or promote informed decision-making.
Level 5	Gender-transformative	Addresses the causes of gender-based health inequities by including ways to transform harmful gender norms, roles, and relations. The objective of such programmes is often to promote gender equality and foster progressive changes in power relationships between women and men.		Designed to promote empowering reproductive and sexual agency and bodily autonomy through postpregnancy contraception by transforming gender norms and relations, including those related to the relationship between sexuality and reproduction, addressing gender-based discrimination and promoting informed decision-making.

Source: Prepared by the authors based on WHO’s Gender Responsive Assessment Scale^16^ and Coates and Allotey^2^ 2023.

We used the acronyms shown in table 2.

**Table 2:** List of acronyms

ANC	Antenatal care	MCH	Maternal and child health
BCS	Balanced Counselling Strategy	MOH	Ministry of Health
CHW	Community health worker	NGO	Non-governmental organisation
FIGO	International Federation of Gynecology and Obstetrics	PAC	Postabortion care
FP	Family planning	PAFP	Postabortion family planning
IPP	Immediate postpartum	PNC	Postnatal care
IMCH	Integrated Maternal and Child Health	PPFP	Postpartum family planning
IUD	Intrauterine device	PPIUD	Postpartum intrauterine device
L&D	Labour and delivery	RMNCH	Reproductive, maternal, newborn and child health
LAM	Lactation amenorrhea method	SRH	Sexual and reproductive health
LARC	Long-acting reversible contraceptive	WHO	WHO

### Patient and public involvement

There was no patient or public involvement in this study.

## Results

**Figure 1:**
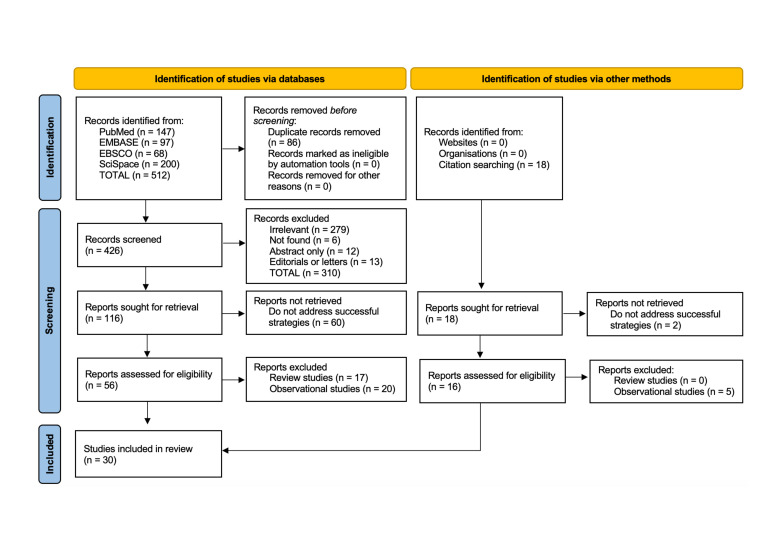
PRISMA flow diagram. Source: Prepared by the authors following the PRISMA 2020 statement.^11^ PRISMA, Preferred Reporting Items for Systematic Reviews and Meta-Analyses.

The PRISMA flow chart (figure 1) shows the identification, screening and inclusion process of the 30 implementation, evaluation and experimental studies retained. 27 studies were conducted in a single country and 3 in multiple countries. Combined, the studies were conducted in Afghanistan (1), Bangladesh (1), Benin (1), Chad (2), China (1), Côte d’Ivoire (1), the Democratic Republic of the Congo (1), Djibouti (1), Ethiopia (3), Ghana (2), Kenya (2), Malawi (1), Mali (1), Nepal (3), Niger (1), Nigeria (2), Pakistan (1), Rwanda (2), Senegal (1), Somalia (1), South Africa (1), Spain (1), Sri Lanka (1), Tanzania (3), Togo (1) and the USA (4). All the studies were published in English.

### Level of gender responsiveness

In table 3, we present the 30 studies according to the level of gender responsiveness reported, starting with level 5, in chronological order of publication. Four studies reported gender-transformative strategies (level 5), 24 were gender-specific (level 4), and 2 were gender-sensitive (level 3). Therefore, none of the strategies hindered the achievement of reproductive health and rights. All the strategies included women and girls, and none were explicitly targeted at gender-diverse people capable of childbearing. Additional information for each implementation, evaluation and experimental study is available in online supplemental annex 1; a summary of the review and observational studies not included in the scoping review is in online supplemental annex 2.

**Table 3:** Level of gender-responsiveness of studies that report successful strategies in expanding postpregnancy contraception

Reference and year	Country	Target population	Description of intervention or strategy
Level 5—Gender-transformative			
Tawfik *et al*^17^ (2014)	Afghanistan	Postpartum women	Integrates FP into postpartum care by providing counselling and addressing each woman’s reproductive intentions in private spaces. Involved husbands and mothers-in-law with the aim of actively challenge power dynamics and promoting women’s reproductive autonomy.
Curry *et al*^18^ (2015)	Chad, the Democratic Republic of the Congo, Djibouti, Mali, and Pakistan	Women of reproductive age and women receiving postabortion care	Focuses on improving FP in crisis-affected regions. Engages men and women in discussions, promotes informed contraceptive decision-making by women and addresses gender relations.
Samuel *et al*^19^ (2016)	Ethiopia	Women receiving abortion care	Employs a rights-based approach to postabortion FP, emphasising women’s reproductive autonomy and informed contraceptive decision-making.
Mbehero *et al*^20^ (2021)	Kenya	Women receiving abortion care	Addresses the provision of safe abortion, postabortion care and contraception, focusing on women’s health services and youth-friendly services, promoting sexual and reproductive rights and challenging gender norms.
Level 4 – Gender-specific			
Adanikin *et al*^21^ (2013)	Nigeria	Pregnant women in the third trimester	Focuses on the specific needs of pregnant women, offering tailored contraceptive counselling and promoting informed contraceptive decision-making. It acknowledges gender-specific reproductive roles but does not provide information on how they addressed underlying gender norms or power relations
Cooper *et al*^22^ (2014)	Bangladesh	Postpartum women	Focuses on the specific needs of postpartum women to promote contraceptive uptake and informed contraceptive decision-making. It acknowledges gender-specific reproductive roles and involves husbands and mothers-in-law but does not challenge underlying gender norms or power relations.
Huang *et al*^23^ (2014)	China	Women receiving childbirth care	Focuses on the specific needs of rural-to-urban migrant women regarding postpartum contraception. The intervention provides free contraceptive counselling and a choice of methods, which helps to overcome financial barriers and empowers single and married women to make informed decisions about their reproductive health but does not actively seek to transform gender relations or norms.
Pleah *et al*^24^ (2016)	Benin, Chad, Côte d’Ivoire, Niger, Senegal, and Togo	Women during pregnancy, labour and immediate postpartum	Targets specific needs of postpartum women for IUD services, addresses cultural barriers, and promotes informed contraceptive decision-making, but does not substantially challenge underlying gender norms or roles.
Chukwumalu *et al*^25^ (2017)	Somalia	Women receiving postabortion care	Focuses on service provision and informed contraceptive decision-making without actively challenging prevailing gender norms.
Gbagbo and Elzohry *et al*^26^ (2018)	Ghana	Women receiving postabortion care	Focuses on increasing postabortion contraception through a comprehensive model that relies on informed contraceptive decision-making but does not explicitly address gender relations or aim to transform them. While the study acknowledges the presence of partner pressure, it does not explicitly analyse how these factors might be impacting the programme’s effectiveness or propose specific strategies to address them.
Ingabire *et al*^27^ (2018)	Rwanda	Women during pregnancy, labour, immediate postpartum, infant vaccination visits, or in the community	Implements a multilevel intervention for postpartum IUD services involving male partners, but primarily focuses on increasing women’s contraceptive uptake without fundamentally changing gender dynamics.
Lori *et al*^28^ (2018)	Ghana	Pregnant women	Increases FP use through group antenatal care, promoting women’s informed contraceptive decision-making, without directly challenging gender norms.
Torres *et al*^29^ (2018)	United States	Postpartum women immediately after giving birth to a premature infant	Enhances knowledge and use of postpartum LARC methods, focusing on women’s informed contraceptive decision-making, but without addressing underlying causes of gender roles or relations.
Wendot *et al*^30^ (2018)	Kenya	Women receiving safe abortion and postabortion care	Improves quality of postabortion FP counselling and method uptake by using BCS (which promotes informed decision-making), targeting women’s informed contraceptive decision-making without actively addressing gender power dynamics. While the study acknowledges the potential impact of gender dynamics (by asking for relationship status and partner support for contraceptive use), it does not explicitly analyse how these factors might be impacting women’s decisions regarding postabortion FP.
Karra *et al*^31^ (2019)	Sri Lanka	Women receiving childbirth care	Increases counselling rates and informed contraceptive decision-making but focuses on women’s health services without challenging underlying gender norms or how these norms might be impacting women’s experiences with the programme or their choices regarding postpartum IUD.
Mossie *et al*^32^ (2019)	Ethiopia	Postpartum women	Focuses on tracking postpartum FP counselling and informed contraceptive decision-making through the continuum of care. The study acknowledges couple or family discussion of PPFP as an input to uptake but does not analyse how gender dynamics or social norms might be impacting women’s experiences with PPFP or their decision-making processes.
Pradhan *et al*^33^ (2019)	Nepal	Women in the immediate postpartum	Increases counselling and uptake of postpartum IUD, increasing women’s contraceptive knowledge without explicitly analysing how gender dynamics or social norms might be impacting women’s experiences with the programme or their choices regarding PPIUD.
Stephens *et al*^34^ (2019)	Tanzania	Women receiving postabortion care	Focuses on expanding postabortion contraceptive coverage with balanced counselling strategy (which promotes informed contraceptive decision-making) and respectful care but does not actively seek to transform gender relations.
Haider *et al*^35^ (2020)	USA	Postpartum women within 4.5 months of giving birth	Offers colocated contraceptive services with infant care to enhance informed contraceptive decision-making. Considers marital status or living arrangements, acknowledging the potential impact of gender dynamics but does not develop actions to change gender norms or relations.
Huber-Krum *et al*^36^ (2020)	Nepal	Women during pregnancy, early labour and immediate postpartum	Focuses on training providers in counselling and postpartum IUD insertion, promoting informed contraceptive decision-making. Acknowledges gender norms and social pressure to have children but does not analyse how these might be impacting women’s experiences with the programme or their decision-making processes.
Lacy *et al*^37^ (2020)	USA	Women seeking immediate postpartum or postabortion LARC	Increases access to immediate postpartum LARC, focusing on women’s health needs and informed contraceptive decision-making but does not analyse how gender dynamics or social norms might be impacting women’s experiences with immediate postpartum LARC or their decision-making processes.
Pearson *et al*^38^ (2020)	Tanzania	Women during pregnancy and after admission for delivery	Increases counselling and choice of postpartum IUD, targeting women’s informed contraceptive decision-making. Considers marital status or living arrangements, acknowledging the potential impact of gender dynamics, but does not develop actions to change gender norms or relations.
Reyes-Lacalle *et al*^39^ (2020)	Spain	Pregnant women	Enhances contraceptive use through additional counselling, focusing on women’s informed contraceptive decision-making. Considers the partner’s involvement and the marital or civil union status and acknowledges the study limitation that intimate partner violence was not analysed. However, it does not analyse how gender dynamics or social norms might be impacting women’s experiences with contraception or their decision-making processes.
Sitrin *et al*^40^ (2020)	Ethiopia	Pregnant women	Integrates postpartum FP into community-level services, focusing on women’s contraceptive informed contraceptive decision-making and use. Considers marital status but does not analyse how gender dynamics or social norms might be impacting women’s experiences with the programme or their decision-making processes.
Wu *et al*^41^ (2020)	Nepal	Women in the 8th month of pregnancy and at 1, 5 and 10 months post partum	Increases contraceptive use through balanced counselling strategy (which promotes informed contraceptive decision-making), community-based counselling, targeting women’s needs without explicitly addressing gender dynamics.
Espey *et al*^42^ (2021)	Rwanda	Women during pregnancy, early labour and delivery, and infant vaccination visits	Implements a multilevel intervention to increase uptake of postpartum LARC methods, promoting informed contraceptive decision-making and male engagement but without seeking to transform gender norms and relations.
Karra *et al*^43^ (2022)	Malawi	Women during pregnancy or within 6 months post partum	Increases contraceptive use and birth spacing, targeting women informed contraceptive decision-making. As acknowledged in the study limitations, while the study provided guidance to women on ways to involve their husband or male partner via counselling and dialogue, leaving up to the women how much she wanted to involve him in any part of the counselling or intervention, it did not specifically address the social norms concerning community or partner perspectives on FP, alleging that it would have required a larger, cluster randomised, multiarmed trial.
Ijarotimi *et al*^44^ (2023)	Nigeria	Postpartum women attending infant vaccination clinics.	Integrates FP education with infant vaccination, targeting women’s informed contraceptive decision-making. Considers the marital status without addressing the underlying gender dynamics that might play a role in contraceptive choices.
Level 3—Gender-sensitive			
Melnick *et al*^45^ (2016)	USA	Vulnerable, low-income, first-time mothers at risk for unintended and short-interval pregnancies	Focuses on decreasing women’s gaps in use of effective methods after childbirth to reduce the incidence of unintended and short-interval pregnancies but does not address informed contraceptive decision-making nor how gender dynamics might be impacting women’s experiences with the programme.
Furey and Fiander *et al*^46^ (2019)	Tanzania and South Africa	Women admitted for childbirth	Focuses on improving the uptake and choice of postpartum FP but does not address informed contraceptive decision-making nor how gender dynamics might be impacting women’s experiences with the programme.

FPfamily planningIUDintrauterine deviceLARClong-acting reversible contraceptivePPFPpostpartum family planningPPIUDpostpartum intrauterine device

### Characteristics of gender-responsive strategies that reported success in expanding postpregnancy contraception

The studies that reported gender-transformative strategies successful in expanding postpregnancy contraception^17^,^20^ shared the goal of improving the quality of contraception counselling and service provision. They shared a commitment to empowering women with the autonomy to make informed decisions about postpregnancy contraception through (a) delivering personalised counselling that respected each woman’s reproductive goals and ensured privacy during these discussions; (b) integrating the cultural and familial context of women’s health decisions and (c) promoting a rights-based approach that prioritised informed consent and defended women’s reproductive rights. These interventions were delivered in contexts of quality improvement strategies that integrated contraception services into the continuum of postpregnancy care and that offered women continuous access to information.

In Afghanistan,^17^ the intervention involved integrating family planning (FP) into postpartum care using quality improvement approaches focused on improving the quality of counselling and turning the process user-centred. This included tailoring the counselling process to address each woman’s specific reproductive intentions and contraceptive needs, finding private spaces for postpartum family planning (PPFP) counselling, and involving husbands and mothers-in-law in counselling. Follow-up calls from FP counsellors clarified contraceptive methods and underscored essential FP messages. These enhanced approaches successfully integrated FP into postpartum care, resulting in more women receiving counselling on postpartum FP and acquiring their chosen method before leaving the hospital. The strategy addressed gender-based factors by involving husbands and mothers-in-law in FP counselling. Before the strategy, the implementation team had identified that husbands were not allowed in the maternity ward and that some women wanted to consult with their husbands or mothers-in-law before making their decision on contraception use. Creating private spaces for postpartum FP counselling where the husband or the mother-in-law could participate, in person or via mobile phone, was a gender-transformative approach that contributed to meeting women’s needs and a rise in joint decision-making on SRH needs.

In Chad, the Democratic Republic of the Congo, Djibouti, Mali and Pakistan,^18^ the choice of contraceptives among women of reproductive age and those receiving postabortion care varied by country, with long-acting reversible contraceptives (LARCs) being more prevalent in specific regions and other methods like oral contraceptives and injectables being dominant in others. The study discussed the role of community norms related to gender and fertility in implementing FP services. It emphasised the importance of engaging men and women in discussions about rights, fertility and contraception at the local level. The strategy also highlighted the role of religious leaders in promoting modern contraception, indicating an understanding of the gender dynamics within religious contexts. Improving FP accessibility and education in crisis-affected regions involved several components: (a) conducting health facility assessments to identify infrastructure gaps, reviewing the presence and condition of basic needs and reequipping facilities with essential supplies and equipment; (b) providing comprehensive training to healthcare providers, using materials from Jhpiego and the Population Council; this includes both theoretical sessions and practical training; (c) engaging with facility teams and community leaders to ensure a clean, efficient, and respectful environment and regular supervisory visits to assess clinic conditions, stock levels and data consistency; (d) ensuring a steady supply of contraceptive methods and related items by both procuring necessary items and working to strengthen supply chain management in collaboration with government systems and (e) raising awareness at the community level through various means such as radio, participatory theatre and group dialogues, and engaging religious leaders across faiths to raise awareness and ensure women’s rights to access health services. The key to community mobilisation’s success was motivating and involving the entire community to recognise the advantages and possibilities in planning and spacing childbirth and facilitating access for women to use services.

In Ethiopia,^19^ the strategy consisted of a package of interventions for women seeking abortion care that promoted a rights-based approach to informed consent and decision-making. The strategy improved the quality of PAFP counselling, increased the contraceptive method mix, emphasised and prioritised respect for women’s reproductive autonomy in the provision of PAFP services, promoted youth-friendly services, ensured providers’ commitment to the sexual and reproductive rights of young women, promoted privacy and confidentiality during service delivery and strengthened community outreach through health extension workers. These strategies resulted in an increase in postabortion choice of a contraceptive method and a greater proportion choosing the more effective, long-acting methods.

In Kenya,^20^ health providers were trained on abortion values clarification, attitude transformation, methods of uterine evacuation, management of abortion complications and postabortion contraception. Training and mentorship of providers on PAFP and youth-friendly services, along with community engagement and referrals, helped remove common barriers to care: (a) the project addressed the issue of providers’ hesitation to counsel and administer LARCs to adolescent or youth users through training and mentoring; (b) each facility had separate procedure rooms for conducting medical vacuum aspiration to ensure adequate space and user confidentiality; (c) implemented training and mentorship programmes for mid-level providers on safe abortion, postabortion care and postabortion contraception, with a focus on youth-friendly services; (d) implemented a quality assessment tool based on the WHO’s seven pillars of health systems strengthening and the structures and processes in place to support the domains of quality (safety, timeliness, effectiveness, efficiency, equity and people-centredness); (e) created health facility registers to track postabortion contraceptive uptake; (f) monitored mentees’ clinical competencies through logbooks, tracking their proficiency in LARC insertion and removal and manual vacuum aspiration; (g) monitored the increase in couple counselling for SRH, indicating a rise in joint decision-making on SRH needs; (h) community engagement and referral activities to raise awareness and reduce the stigma associated with abortion and contraception; (i) incorporation of strong SRH rights advocacy partners into the project to build networks and enhance access to contraception and safe abortion; (j) prioritisation of youth-led SRH rights advocacy groups in projects to empower young people to make informed decisions about their SRH; (k) establishment of quality improvement teams at each facility to assess progress and address gaps, fostering a culture of quality and motivation; (l) education of teachers on SRH rights, recognising the impact of teachers on students’ contraceptive use and (m) implemented strategies to increase male involvement in SRH rights, including holding forums for men and mentoring male champions. The increase in couple counselling for SRH indicated a rising normalisation of joint decision-making on SRH needs.

The gender-specific strategies^21^,^44^ focused on informed contraceptive decision-making while recognising the potential impact of gender dynamics on contraceptive use without actively challenging the underlying gender norms or power relations. The most common gender-specific strategies to expand postpregnancy contraception included: (a) enhancing provider–patient interactions through tailored, structured or balanced counselling, such as in Nigeria,^21^ the USA,^29 37^ Kenya,^30^ Tanzania^34^ ; (b) increasing the variety of contraceptive choices to better respond to women’s needs, such as in China^23^ and Somalia^25^; (c) integrating contraceptive education with health services like antenatal visits, such as in Ethiopia where they used a modified Integrated Maternal and Child Health to record the woman’s method choice during those visits,^32^ Nepal,^33 36 41^ Spain,^39^ Ethiopia,^40^ Rwanda^42^ and Malawi^43^; right after admission for delivery services, such as in Tanzania^38^; during infant vaccinations, such as in Rwanda,^27^ Ghana^28^ and Nigeria^44^; during the first year postpartum, such as in China^23^; and other maternal and child health services, such as in the USA^29 35^; (d) introducing competency-based training for healthcare providers, such as in Benin, Chad, Côte d’Ivoire, Niger, Senegal and Togo,^24^ Somalia,^25^ Ghana^26^; (e) employing storytelling with relatable content, such as in Bangladesh^22^ and Ghana^28^ or showing informational videos in hospital waiting areas, such as in Sri Lanka^31^ and Nepal.^33^ Community engagement, particularly involving religious and community leaders, was critical in addressing cultural and gender norms, as shown in Bangladesh,^22^ Benin, Chad, Côte d’Ivoire, Niger, Senegal, Togo^24^ and Somalia.^25^

Finally, the gender-sensitive strategies reported in one study conducted in the USA^45^ and one in Tanzania and South Africa^46^ were aimed at either increasing the uptake and choice of postpartum contraception through education and training of midwives, nurses and non-specialist doctors on counselling and provision or by enhancing an existing nurse home visiting programme. Although these studies indicated gender awareness, they did not address the process of informed contraceptive decision-making, the impact of gender dynamics on women’s experiences with the programme or the importance of creating supportive environments that respect and enhance bodily autonomy.

Among the studies reviewed, we did not find indicators that measure and evaluate gender-related barriers and promote bodily autonomy. Online supplemental annex 3 shows a list of indicators based on the strategies that measure and evaluate the rights-based counselling and provision of postpregnancy contraception.

### Key components of gender-transformative strategies that can be adapted to different contexts or scaled up

Based on the four gender-transformative studies,^17^,^20^ box 1 shows the multifaceted approach needed to successfully implement gender-transformative interventions in postpregnancy contraception across different cultural and regional contexts. Details from all the studies are available in online supplemental annex 1.

Box 1 **Key components of gender-transformative strategies that can be adapted to different contexts or scaled up**

**A. Training, capacity building, continuity of care and supply chain management**

1. Training healthcare staff in user-centred and rights-based contraception counselling during pregnancy and postpregnancy to ensure informed decision-making and improve service delivery.
2. Training and mentorship programmes for healthcare providers on safe abortion, postabortion care and contraception, with a focus on youth-friendly services, to sustain quality improvement strategies that support gender-transformative strategies.
3. Monitoring and tracking the clinical competencies of providers providing gender-transformative care, for example, through logbooks.
4. Creating private spaces for counselling to ensure privacy and comfort.
5. Ensuring follow-up to address each woman’s specific reproductive intentions and contraceptive needs, for example, through follow-up calls to answer questions about contraceptive methods.
6. Implementing supply chain management approaches that consider local challenges to sustain quality improvement plans that support gender-transformative strategies.
7. Monitoring of health systems quality issues, including procurement and supply chains, to ensure women have access to a wide range of contraceptive methods to increase the proportion of postpregnancy women leaving the facility with their method of choice.

**B. Counselling and empowerment**

1. Tailoring the counselling process to address each woman’s specific reproductive intentions and contraceptive needs and prioritise autonomous decision-making.
2. Involving key family members, such as partners and mothers-in-law, in counselling sessions to challenge existing power dynamics and promote women’s reproductive autonomy and equality in reproductive health decision-making.
3. Increasing couple’s counselling for sexual and reproductive health (SRH) to normalise joint decision-making on SRH needs.

**C. Community engagement, mobilisation and advocacy**

1. Engaging people of all genders in local discussions about rights, fertility and contraception to address and transform cultural and gender-based norms and relations.
2. Developing community mobilisation, engagement and referral activities through radio, participatory theatre and group dialogues, involving religious leaders across faiths and local leaders to discuss the role of community norms related to gender and fertility and raise awareness and support women’s rights to access health services.
3. Increasing male involvement in discussions about sexual and reproductive health and rights, fertility and contraception, including holding forums for men and mentoring male champions, to address and transform cultural and gender-based norms and relations at the local level.
4. Prioritisation of youth-led SRH rights advocacy groups in projects to address and transform cultural and gender-based norms and relations.
5. Incorporation of strong SRH rights advocacy partners in the interventions to build networks and enhance access to contraception and safe abortion.
6. Engaging in dialogue with local community groups, especially women’s organisations to better understand local gender norms and women’s reproductive needs.

Note: Prepared by the authors based on gender-transformative studies.^17^,^20^

### Factors associated with using digital tools to promote postpregnancy contraceptive use

Of the 30 studies included in the review, 10 referred to the use of digital tools to promote postpregnancy contraceptive use.^2427 32 34^,^39 41^ Two studies conducted in Nepal and one in Spain reported that digital technology in healthcare improved service integration and facilitated the dissemination of contraceptive information.^36 39 41^ In Tanzania, mobile applications and digital counselling tools were instrumental in training healthcare workers, giving them access to the latest information and techniques to meet users’ needs effectively.^34^ Digital data collection and management systems enhanced the continuity of care and follow-up of women users of contraception. For example, in Benin, Chad, Côte d’Ivoire, Niger, Senegal and Togo, digital tools helped identify postpartum intrauterine device (PPIUD) complications.^24^ In Ethiopia, digital health records helped improve service integration and continuity of care, track PPFP and facilitate decision-making and timely adoption of contraceptives.^32^ In Spain, women received reminders to consult contraception information during pregnancy.^39^ In Nepal, community health workers used mobile applications to individualise the counselling based on women’s responses to the balanced counselling strategy questions and to follow-up at subsequent visits on initiation, barriers to access and continuation.^41^ Finally, tablets and software for data input were used to collect data in Rwanda,^27^ Tanzania,^38^ the USA,^35 37^ Spain^39^ and Nepal.^41^

## Discussion

We reviewed 30 studies that met all the inclusion criteria. All but two studies emphasised the need to provide a supportive environment that respects and enhances women’s bodily autonomy. The gender-transformative strategies reported in four of the studies shared a commitment to empowering women with the autonomy to make informed decisions about postpregnancy contraception. They achieved this through quality improvement strategies that seamlessly integrated contraception services into the continuum of postpartum and postabortion care. Providers delivered personalised counselling that respected each woman’s reproductive goals and ensured privacy during these discussions. The initiatives included follow-up support to offer women continuous access to information and reinforce contraceptive practices. These efforts promoted a rights-based approach, prioritising informed consent and defending women’s reproductive rights. Women often chose LARC, reflecting a preference for more reliable and enduring contraception methods when they received comprehensive information. These programmes acknowledged and integrated the cultural and family context of women’s health decisions by involving husbands and mothers-in-law, not as vehicles to increase postpregnancy contraception but to challenge existing power dynamics and promote women’s reproductive autonomy and equality in reproductive health decision-making. The implementation of these strategies, carefully adapted to each context, underscores the effectiveness of a comprehensive, multilayered approach in transforming health interventions related to gender dynamics. By tackling both service delivery and broader social elements like gender norms and community standards, the findings from the gender-transformative strategies illustrate the potential for significant advancements in expanding postpregnancy contraception.

The gender-specific studies focused on informed contraceptive decision-making. The studies demonstrated that multiple counselling sessions during antenatal care, tailored counselling during the third trimester, the free provision of contraception integrated into childbirth and abortion care, a range of options in contraceptive methods and follow-up visits were crucial in ensuring the informed contraceptive decision-making process. Training healthcare providers in insertion techniques and providing informed consent and rights-based counselling was pivotal in increasing the informed uptake of postpregnancy LARC methods and, specifically, PPIUD. Community engagement strategies that included the participation of religious and community leaders were instrumental in facilitating women’s access to health facilities for postabortion care services. The strategies also acknowledged the need for comprehensive monitoring and evaluation frameworks for data-driven programmatic decisions, engagement of key stakeholders from the initial stages and the importance of youth-friendly services. While they recognised the potential impact of gender dynamics on contraceptive use and some interventions involved key family members in the counselling process, some strategies did not actively challenge the underlying gender norms or power relations.

Although several scoping and systematic reviews have addressed strategies that increase postpregnancy uptake of contraception around the world,^47^,^55^ this is the first scoping review of interventions that promote bodily autonomy when addressing gender-related barriers to scaling up and sustaining postpregnancy contraception. These comprehensive strategies underscore the intricate relationship between health systems and social structures, showcasing how public health interventions prioritising SRH can act as transformative tools to advance gender equality, as other authors have argued.^3 10^ By improving access to postpregnancy and postabortion contraception, fostering culturally sensitive and rights-based counselling and engaging key community stakeholders, these approaches enhance women’s autonomy and facilitate equitable decision-making processes. Such interventions exemplify how strengthening health systems through quality improvement, community mobilisation and inclusive engagement can empower individuals, bridge gender inequities and enable greater access to education and reproductive choices, ultimately contributing to broader social transformation.

The review has allowed us to identify key elements of gender-transformative postpregnancy contraceptive strategies, with some important components that can be adapted to diverse contexts. Personal counselling emerged as a common thread across many interventions within a framework of a user-centred approach. Similarly, community dialogue and involvement of advocacy organisations, especially women’s organisations, are crucial to understanding local gender norms and women’s reproductive needs. Both are fundamental to delivering the individualised care required to promote bodily autonomy. However, for bodily autonomy to be meaningfully realised, other elements are also vital. With some notable exceptions, few strategies noted the wider health system context, such as procurement and supply chains to enable providers to meet women’s contraceptive needs, institutionalised quality assurance mechanisms stressing gender transformative approaches and ensuring an enabling health policy and legal environment.

Understanding cultural and familial contexts is another key element. The precise intent of strategies tailored to context is significant to ensure that they do not inadvertently reinforce unequal power relations, rather than advancing women’s bodily autonomy. It is, therefore, especially significant that although the review allowed us to generate a list of results indicators to measure and evaluate the rights-based counselling and provision of postpregnancy contraception, we did not find indicators that measure and evaluate gender-related barriers and promote bodily autonomy. While success is still only defined according to contraceptive access and uptake, it cannot be explicitly guaranteed that any intervention will be delivered in such a manner that it will meaningfully promote bodily autonomy.

We recommend that future research urgently focus on developing and validating indicators that specifically address gender-related barriers and promote bodily autonomy so that contraceptive uptake is not mistakenly understood as a sole proxy for bodily autonomy, but rather as a dual goal. There is a need for more comprehensive strategies that integrate these indicators—and thus approach and goals—into practice, ensuring that postpregnancy contraception programmes empower women and girls, with the tools, knowledge and resources to make informed decisions that align with their life goals.

## supplementary material

10.1136/bmjgh-2024-016638 online supplemental file 1

## Data Availability

All data relevant to the study are included in the article or uploaded as supplementary information.
