# Supplementary material for: Successful strategies that address gender-related barriers and promote bodily autonomy within efforts to scale up and sustain postpregnancy contraception: a scoping review
Source: BMJ Glob Health. 2025 Feb 16;10(2):e016638. doi: 10.1136/bmjgh-2024-016638 (PMC11831305; doi:10.1136/bmjgh-2024-016638)
Supplement: online supplemental file 1 [file bmjgh-10-2-s001.pdf]

Annex 1: Implementation, evaluation, and experimental studies identified in the scoping review by the level of gender responsiveness and chronological order (2013-2023)

There were 30 implementation, evaluation, and experimental studies. Twenty-seven were conducted in a single country and 3 in multiple countries. Combined, the studies were conducted in: Afghanistan (1), Bangladesh (3), Benin (2), Chad (2), China (1), Côte d'Ivoire (1), the Democratic Republic of the Congo (1), Djibouti (1), Ethiopia (3), Ghana (2), Kenya (2), Malawi (1), Mali (1), Nepal (3), Niger (1), Nigeria (2), Pakistan (1), Rwanda (2), Senegal (1), Somalia (1), South Africa (1), Spain (1), Sri Lanka (1), Tanzania (3), Togo (1), and United States (4). All the studies were published in English. **Table A1** shows the study characteristics of each publication by year, country, population studied, and methodological design, followed by the evidence they provide in the selected categories, when available: strategies that report success, intervention results, adaptable key components, strategies adapted in other settings, results indicators, and digital tools and, finally, the level of gender responsiveness.

Table A1: Implementation, evaluation, and experimental studies identified in the scoping review by level of gender responsiveness and chronological order (2013-2023)

Reference and year	Country	Population (and sample size when applicable)	Methodological design
**Implementation, evaluation, and experimental studies (N=29)**			
**Level 5 of gender responsiveness: gender-transformative (N=4)**			
Tawfik, Y., et al. (2014) (1)	Afghanistan	The population studied were 580 postpartum women in Isteqlal and Malalai public hospitals in Afghanistan who received FP counseling and decided to use FP methods. The study also involved husbands and mothers-in-law in the counseling process.	The study used modern quality improvement (QI) approaches to analyze barriers and identify solutions for integrating FP into postpartum care. The QI process involved measuring a performance gap, understanding the causes of the gap, testing, planning, and implementing interventions to close the gap, studying the effects of the interventions, and planning additional corrective actions in response. The study also used collaborative improvement, a collective improvement activity that unites the efforts of a number of teams to work together to rapidly achieve significant improvements in processes, quality, and efficiency of a specific area of care. The study emphasized on analyzing the systems and processes of delivering services and testing changes to obtain better results.

Strategies that report success

- The intervention involved integrating FP into postpartum care using quality improvement approaches.
- These focused on improving the quality of counseling and turning the process user-centered, such as tailoring the counseling process to address each woman's specific reproductive intentions and contraceptive needs, finding private spaces for PPFP counseling, and involving husbands and mothers-in-law in counseling.
- Follow-up calls from FP counselors provided clarity on contraceptive methods and underscored essential FP messages.
- These enhanced approaches successfully integrated FP into postpartum care, resulting in more women receiving counseling on postpartum FP and acquiring their chosen method before leaving the hospital.
- The study addressed gender-based factors by involving husbands and mothers-in-law in FP counseling in person or via mobile phone.
- Creating private spaces for postpartum FP counseling was another gender-sensitive approach that contributed to the program's success.

Intervention results

- By the end of the project, 95% of postpartum women receiving FP counseling at the hospital left with their preferred method.

Adaptable key components

- Modern quality improvement approaches focused on analyzing service delivery systems and processes for better results.
- Involving key family members in counseling sessions to address cultural and gender-based barriers.
- Creating private spaces for counseling to ensure privacy and comfort.
- Training of healthcare staff in user-centered FP counseling to improve service delivery.
- Use of follow-up calls to reinforce key messages and answer questions about contraceptive methods.
- Promoting collaborative improvement, a collective activity that unites multiple care teams to improve processes, quality, and efficiency significantly.
- User-centered perspective that focuses on the needs and expectations of the user within their community.

Results indicators

- Increase in the percentage of women receiving postpartum and post-abortion contraception counseling and their preferred method before discharge.
- Significant decrease in self-reported pregnancy up to 18 months post-discharge compared with women receiving routine services.
- The difference in outcomes between intervention and control groups at 6, 12, and 18 months follow up.

Level of gender responsiveness

- Level 5 (Gender-transformative). Integrates FP into postpartum care by providing counseling and addressing each woman's reproductive intentions in private spaces. It involves husbands and mothers-in-law with the aim to actively challenge power dynamics and promote women's reproductive autonomy.

Curry, D. W., et al. (2015) (2)	Chad, the Democratic Republic of the Congo, Djibouti, Mali, and Pakistan.	Government health workers, nurses, midwives, community leaders, women's associations, health committees, and Lady Health Visitors, depending on the specific country context. The program was active in 79 facilities across 8 districts in 5 countries, serving a population of 2,428,145, with 698,053 women of reproductive age.	The paper describes the Supporting Access to Family Planning and Post-Abortion Care in Emergencies (SAFPAC) initiative, an implementation program and best practices for delivering high-quality FP services with a comprehensive range of contraceptive methods in crisis-affected settings. It discusses various strategies such as competency-based training, supply chain support, systematic supervision, and community mobilization. The paper also emphasizes the use of data to drive program improvements.

Strategies that report success

- The choice of contraceptive varied by country, with LARCs being more popular in certain regions, and other methods like oral contraceptives and injectables being dominant in others.
- Improving FP accessibility and education in crisis-affected regions involved:
  - Facility assessments and stocking: conducting health facility assessments to identify infrastructure gaps, reviewing the presence and condition of basic needs, and reequipping facilities with essential supplies and equipment.
  - Competency-based training: providing comprehensive training to health care providers, using materials from Jhpiego and the Population Council. This includes both theoretical sessions and practical training.
  - Supervision and quality improvement: engaging with facility teams and community leaders to ensure a clean, efficient, and respectful environment. Regular supervisory visits are conducted to assess clinic conditions, stock levels, and data consistency.
  - Supply chain management: ensuring a steady supply of contraceptive methods and related items. This involves both procuring necessary items and working to strengthen supply chain management in collaboration with government systems.
  - Community mobilization: raising awareness at the community level through various means such as radio, participatory theater, and group dialogues. Engaging religious leaders across faiths to raise awareness and ensure women's rights to access health services.
- Gender-based factors addressed: the paper discusses the role of community norms related to gender and fertility on the implementation of FP services. It emphasizes the importance of engaging men and women in discussions about rights, fertility, and contraception at the local level. The paper also highlights the role of religious leaders in promoting modern contraception, indicating an understanding of the gender dynamics within religious contexts. The key to the success of community mobilization lied in motivating and involving the entire community to recognize the advantages and possibilities not just in planning and spacing childbirth, but also in facilitating access for women to utilize services.

Intervention results

- Between July 2011 and December 2013, SAFPAC exceeded its target by reaching 52,616 new contraceptive users—surpassing the target of 36,002 new users—with 61% opting for a long-acting reversible contraceptive (LARC).

Adaptable key components

- The community mobilization approach, especially involving religious leaders, has been successful in diverse contexts.
- Competency-based training can be adapted to different contexts and scaled up.
- Supply chain management techniques and strategies that can be adapted to different regions, considering local challenges.

Level of gender responsiveness

- Level 5 (Gender-transformative). Focuses on improving FP in crisis-affected regions and engaging men and women in discussions, promotes informed contraceptive decision-making by women, and addresses gender relations.

Samuel, M., et al. (2016) (3)	Ethiopia	The study population consisted of women seeking abortion care in Ethiopia's Southern Nations, Nationalities, and People's Region (SNNPR). The sample size was a total of 44,682 women who sought abortion care in 101 public health facilities in the region between July 2009 and June 2014. Among these, 34,212 (77%) received safe and legal induced abortions, and the remaining 10,470 women (23%) received postabortion care for complications of unsafe abortions or complicated miscarriages. Most of the women, 60%, were young, with only 40% of women who sought abortion care during this period being older than 24.	This study is an implementation research design that employed a health systems research approach to assess method continuation and discontinuation rates and evaluate the impact of improvements in postabortion family planning (PAFP) services. Data collection was done through extracting information from the abortion services logbook using a data collection form developed for this purpose. The data collected included information on the type of abortion care, abortion technology, age category, gestational age of the pregnancy, and postabortion contraceptive method chosen by each woman. The study period was from July 2009 to June 2014.

Strategies that report success

- The study used a package of interventions for women seeking abortion care that promoted a rights-based approach to informed consent and decision-making. The strategy improved the quality of PAFP counseling, increased the contraceptive method mix, prioritized respect for women's reproductive autonomy in the provision of PAFP services, promoted youth-friendly services, ensured providers' commitment to the sexual and reproductive rights of young women, promoted privacy and confidentiality during service delivery, and strengthened community outreach through health extension workers.
- The study concluded that the package of interventions introduced in service expansion during the past half-decade tested the impact of focused attention to postabortion service quality on the uptake of contraceptive methods, particularly on the choice of LARC.
- The study also noted that the program had recently begun a phase-out strategy aimed at ensuring self-reliance among high-performing facilities to sustain the delivery of comprehensive abortion care without routine NGO support.

Intervention results

- The comprehensive strategy improved PAFP services and increased the uptake of effective LARC. The proportion of post-abortion women who left the facility with a contraceptive method increased from 58% in 2010 to 83% in 2014. Among the 36,051 post-abortion women who accepted contraception between 2010 and 2014, short-acting hormonal methods (injectables and oral contraceptives) declined from 98% to 45%, and LARC increased from 2% to 55%.

Adaptable key components

- PAFP interventions that promote a rights-based approach to informed consent and decision-making improve women's knowledge of contraception benefits and increase the uptake of contraceptive methods immediately after abortion.
- Introducing a wider range of contraceptive methods in PAFP significantly increases the proportion of women leaving the facility with a method.
- Comprehensive PAFP counseling promotes the choice of more effective methods as women learn more about less common contraceptive options.
- A rights-based approach to informed consent and decision-making.
- Training healthcare providers in LARC insertion.
- Improving the quality of FP counseling.

Level of gender responsiveness

- Level 5 (Gender-transformative). Employs a rights-based approach to postabortion FP, emphasizing women's reproductive autonomy and informed contraceptive decision-making.

Mbehero, F., et al. (2021) (4)	Kenya	Women in five communities who attended 80 public and private health facilities in Homa Bay, Kisii, Kisumu, Migori, and Siaya counties in South West Kenya between March 2018 and August 2020. N=20,683. The project involved training health providers, community health volunteers, and youth peer providers.	Monitoring and evaluation of a multi-pronged intervention that included training and mentorship of mid-level providers on safe abortion, post-abortion care, post-abortion contraception, and youth-friendly services, coupled with community engagement and referral. Quality improvement teams were formed at each facility to assess quarterly progress and address gaps, using a quality assessment tool developed for the project. Newly trained providers were assigned to mentors who provided technical assistance during the project period. The project also focused on community-based demand-creation activities, education, and referrals provided by youth peer providers and community health volunteers.

Strategies that report success

- Health providers were trained on abortion values clarification, attitude transformation, methods of uterine evacuation, management of abortion complications, and post-abortion contraception.
- Training and mentorship of providers on PAFP and youth-friendly services, along with community engagement and referrals, helped remove common barriers to care:
  - The project addressed the issue of providers' hesitation to counsel on and administer LARCs to adolescent or youth users through training and mentoring.
  - Each facility had separate procedure rooms for conducting medical vacuum aspiration to ensure adequate space and user confidentiality.
  - Implementation of training and mentorship programs for mid-level providers on safe abortion, post-abortion care, and post-abortion contraception, with a focus on youth-friendly services.
  - Quality assessment tool based on the WHO's seven pillars of health systems strengthening and the structures and processes in place to support the domains of quality (safety, timeliness, effectiveness, efficiency, equity, and people-centeredness).
  - Standardized health facility registers to track post-abortion contraceptive uptake.
  - Monitoring of the clinical competencies of mentees through logbooks, tracking their proficiency in LARC insertion and removal, and manual vacuum aspiration.
  - Monitoring of the increase in couple counseling for SRH, indicating a rise in joint decision-making on SRH needs.
  - Community engagement and referral activities to raise awareness and reduce the stigma associated with abortion and contraception.
  - Incorporation of strong SRH rights advocacy partners into the project to build networks and enhance access to contraception and safe abortion.
  - Prioritization of youth-led SRH rights advocacy groups in projects to empower young people to make informed decisions about their sexual and reproductive health.
  - Establishment of quality improvement teams at each facility to assess progress and address gaps, fostering a culture of quality and motivation.
- The project recognized the impact of teachers on students' contraceptive use and educated them on SRH rights information.
- The project implemented strategies to increase male involvement in SRH rights, including holding forums for men and mentoring male champions.
- The increase in couple counseling for SRH indicated a rising normalization of joint decision-making on SRH needs.

Intervention results

- Post-procedure contraception among safe abortion and PAC users rose from 74% to 92%, and post-procedure LARC increased from 64 to 86%. The preferred LARC methods were implants, IUDs, and injectables. Two thirds were aged 24 and younger.

Adaptable key components

- Training and mentorship programs for healthcare providers on safe abortion, post-abortion care, and contraception, with a focus on youth-friendly services.
- Community engagement and referral activities to raise awareness and reduce stigma associated with abortion and contraception.
- Incorporation of strong SRH rights advocacy partners into projects.
- Prioritization of youth-led SRH rights advocacy groups in projects.
- Establishment of quality improvement teams at each facility to assess progress and address gaps.

Results indicators

- Percentage of safe abortion and abortion care users who accepted same-day contraception, with a focus on LARC.

Level of gender responsiveness

- Level 5 (Gender-transformative). Addresses the provision of safe abortion, post-abortion care, and contraception, focusing on women's health services and youth-friendly services, promoting sexual and reproductive rights, and challenging gender norms.

Level 4 of gender responsiveness: gender-specific (N=24)

Adanikin, A. I., et al. (2013) (5)	Nigeria	Pregnant women who had reached gestational ages of 28 to 37 weeks during the first three months of the study period. N=216.	The study evaluated an intervention that offered multiple contraceptive counseling sessions during the third trimester of pregnancy to assess its effectiveness in enhancing the postpartum adoption of modern contraceptive methods in comparison with a single counseling session at the sixth week postnatal visit. Pregnant women were randomized to antenatal (ANC) and postnatal counseling groups. The effectiveness of the counseling sessions was evaluated based on the uptake of modern contraception postpartum.

Strategies that report success

- Enhancing interaction between the provider and patient during ANC counseling sessions considerably boosted contraception usage, particularly among those who were initially indecisive.
- The study authors mentioned that the counseling was structured and tailored to meet the needs of couples, primarily when offered during the third trimester. However, the publication did not provide information on what those needs were.

Intervention results

- Women who received ANC counseling displayed a remarkable increase in the use of modern contraceptives six months postpartum compared to those who had postnatal counseling alone (57.4% vs. 35.4%, $p<0.002$).
- The impact of multiple ANC counseling sessions resulted in a rise in the actual postpartum contraceptive use compared to the projected use post-counseling. While 70.3% of women planned to use any family planning (FP) method after ANC counseling, 77.2% eventually did (an increase of 6.9%). In contrast, in the postnatal group, the intention and actual usage remained at 67.7%.

Adaptable key components

- Multiple counseling sessions during ANC can significantly enhance the actual use of contraception, a strategy that can be scaled up.
- Tailored counseling during the third trimester can increase the use of modern postpartum contraception, a strategy that has been successful in diverse settings.

Results indicators

- Intention to use modern or traditional FP methods postpartum.
- Actual use of modern or traditional FP methods postpartum.

Level of gender responsiveness

- Level 4 (Gender-specific). Focuses on the specific needs of pregnant women, offering tailored contraceptive counseling and promoting informed contraceptive decision-making. It acknowledges gender-specific reproductive roles but does not provide information on how they addressed underlying gender norms or power relations.

Cooper, C. M., et al. (2014) (6)	Bangladesh	Postpartum women (n=40) who had given birth in the past year in Sylhet District, Bangladesh. Focus group discussions were held with all the postpartum women, their mothers or mothers-in-law (n=35), and their husbands (n=34).	The study utilized a qualitative research design, specifically in-depth interviews and focus group discussions, to explore knowledge and perceptions about postpartum return to fecundity and the influence of behavior change communication on contraceptive adoption. The study employed the Steps to Behavior Change (SBC) framework, which identifies five stages of change (knowledge, approval, intention, practice, and advocacy) that individuals experience in adopting a new behavior. This framework was used to assess the reported impact of a leaflet and a fictional story called "Asma's Story" on shifts along the behavior change continuum towards postpartum contraceptive uptake. Semi-structured methods of inquiry were used, allowing for the quantification of some qualitative data. This approach facilitated the assessment of respondents' progress across the stages of behavior change and the identification of barriers and successes related to postpartum contraception.

Strategies that report success

- The key component of the strategy was disseminating knowledge through a relatable story in a leaflet format and its integration into a comprehensive program focusing on maternal, newborn, and child health. The story emphasized the unpredictability of pregnancy after childbirth and the importance of optimal pregnancy spacing. The narrative format, with a character many found relatable, was an effective strategy for knowledge dissemination, as evidenced by the wide range of respondents that moved through various stages of the behavior change continuum after exposure. This approach could potentially be adapted to various contexts by crafting culturally relevant narratives for different groups.
- The paper highlighted barriers faced by women, such as opposition from husbands and certain religious beliefs, and the understanding that decisions about contraception are not taken by women alone.
- The study showed that when families understand the advantages of healthy pregnancy spacing, there's a notable increase in both the motivation to use FP and the adoption of modern contraceptive methods. However, husbands commonly cited that the advantages of spacing included the economic benefits for the family, whereas mothers in law often cited the increased ability of women on FP to do household work.
- One significant concern was addressing the unique challenges of women whose spouses work overseas. To address this, the study suggested emphasizing the initiation of a FP method before the husband's return and considering emergency contraception as a contingency.

Intervention results

- The assessment showed high levels of exposure to the leaflet among respondent groups, with a significant majority of postpartum women having heard the story from community mobilizers or CHWs: 93% of the 40 postpartum women reported that the story and leaflet altered their understanding about fecundity and postpartum family planning (PPFP).
- About 32.5% of postpartum women were using a modern contraceptive method at the time of the interview, and 35 out of 40 respondents indicated that the story/leaflet influenced a change in their behavior, which included contraceptive use and sharing the story with others.

Adaptable key components

- Use of narrative stories and leaflets to influence behavior change towards postpartum contraceptive uptake.
- Incorporation of behavior change communication to address perceptions of postpartum return to fecundity and contraceptive adoption.
- Engagement of key influencers in FP decisions, such as husbands and mothers-in-law.
- Application of the SBC framework to assess progress towards adopting a new behavior. The SBC framework provides a means to evaluate an individual's journey towards embracing and maintaining a new behavior. The SBC model outlines five stages an individual typically undergoes when adopting a new behavior: knowledge, approval, intention, practice, and advocacy. Progressing from one phase to the next enhances the probability of both adopting and consistently practicing the behavior. Effective behavior change initiatives support this progression, guiding individuals towards the desired behavior.
- Bridging the gap between intention and action by ensuring proactive linkage to FP services. Opportunities to bridge the gap between intention and action encompass proactive linking of women, whose husbands are abroad, to FP services before their husbands return.
- Other strategies include increased engagement with religious leaders, encouraging more participation of spouses in community sessions, and devising alternative methods to emphasize information about the lactation amenorrhea method (LAM) and the significance of timely transitions.
- Encouraging advocacy among those who have adopted the new behavior to communicate their experiences to others.

Level of gender responsiveness

- Level 4 (Gender-specific). Focuses on the specific needs of postpartum women to promote contraceptive uptake and informed contraceptive decision-making. It acknowledges gender-specific reproductive roles and involves husbands and mothers-in-law but does not challenge underlying gender norms or power relations.

Huang, Y., R., et al. (2014) (7)	China	Rural-to-urban migrant women in Shanghai enrolled during hospitalization for childbirth. N=840.	The study was an intervention design, providing free contraceptive counseling and a choice of methods to participants. Participants were enrolled during hospitalization for childbirth and followed up at 6 weeks and at 3, 6, 9, and 12 months postpartum via scheduled telephone calls and/or clinic visits. The study was conducted at the Pu Jiang Community Health Center and approved by the Ethics Committee of the International Peace Maternal and Child Health Hospital. Data were collected using the Willingness to Participate Questionnaire, which collected demographic, reproductive history, and prenatal health status information. Multivariate logistic regression modeling was used to measure the impact of an intervention on contraceptive use and unintended pregnancies.

Strategies that report success

- The study successfully integrated free FP services into existing childbirth delivery services, addressing the unmet need for FP among rural to urban migrant women. The study identified that supporting services during the first year postpartum are essential to encourage women to continue contraceptive use and reduce the incidence of postpartum unintended pregnancy.

Intervention results

- The intervention cohort initiated contraceptive use earlier (2 months postpartum) compared to the non-intervention cohorts (8.5 and 7.5 months, respectively). At the end of the first year, contraceptive use in the intervention cohort was 97%, compared to 73.6% and 62.9% in the two non-intervention cohorts. Additionally, the incidence of unintended pregnancies was lower in the intervention cohort (2.2 per 100 women-years) compared to the non-intervention cohorts (12.8 and 9.6, respectively).

• The results from the multivariate logistic regression model showed that the use of long-acting contraceptives was linked with being over 30 years of age, having had one or more children before the most recent child, and the most recent child being male.			
**Adaptable key components**<br>• Providing free contraceptive counseling and a choice of methods during hospitalization for childbirth.<br>• Offering further support and counseling at regular intervals postpartum via scheduled telephone calls and/or clinic visits.<br>• Ensuring access to free contraceptive methods during postpartum hospitalization with support of such services over a 1-year postpartum period. and<br>• Making FP services more accessible postpartum, regardless of marriage status.<br>• This approach can be introduced for the benefit of postpartum women and their infants in other impoverished settings or among hard-to-reach populations where access to health care and resources are limited.			
**Level of gender responsiveness**<br>• Level 4 (Gender-specific). Focuses on the specific needs of rural-to-urban migrant women regarding postpartum contraception. The intervention provides free contraceptive counseling and a choice of methods, which helps to overcome financial barriers and empowers single and married women to make informed decisions about their reproductive health but does not actively seek to transform gender relations or norms.			
Pleah, T., et al. (2016) (8)	Benin, Chad, Côte d'Ivoire, Niger, Senegal, and Togo.	ANC, maternity, and postnatal care providers from 5 francophone African countries. N=2,269.	This implementation study focused on introducing and expanding postpartum family planning (PPFP) and postpartum IUD (PPIUD) services, training of healthcare providers, and coordinating between different healthcare services. The study used a pre-post design to evaluate the impact of competency-based training for healthcare providers on the uptake of PPIUD services. The interventions used to increase contraceptive uptake included training healthcare providers in PPIUD insertion and counseling, providing job aids and equipment, and conducting supportive supervision and mentoring. The study also used a quality improvement approach to address barriers to PPIUD uptake and improve the quality of care.
**Strategies that report success**<br>• Competency-based training for healthcare providers, combined with quality improvement approaches, can increase the uptake of PPIUD services and improve the quality of care. This training led to an enthusiastic response from providers, government, and donors for expanding the approach.<br>• The study highlighted the importance of addressing cultural and religious barriers to PPIUD uptake and the need for ongoing data collection and monitoring to ensure the program's sustainability.<br>• The paper emphasizes the importance of partnerships between donors, national MOHs, and other key stakeholders in the success of the initiative, suggesting that collaboration and coordination may be important components of successful strategies. Togo was particularly successful, with a robust training program and a high number of IUD insertions.			
**Intervention results**<br>• The study showed a significant increase in the uptake of PPIUD services in the intervention sites. Of the 15,394 postpartum women counseled about PPFP, 2,269 (14.7%) chose and received the postpartum IUD between 2014 and 2015 in all the countries with data (this excludes Chad, where the study was delayed). This is a substantial increase for a region where the use of PPIUD was less than 1% among married women of reproductive health.<br>• Women who attended ANC were more likely to initiate PPFP, showing the effectiveness of information dissemination during pregnancy.<br>• The study also found that the quality of PPIUD services improved over time, as measured by a reduction in the risk of expulsion associated with post-placental and immediate postpartum insertion.<br>• A significant proportion of PPIUD recipients received a follow-up consultation, indicating successful post-insertion care.<br>• The most common timing for PPIUD insertion was during cesarean delivery, demonstrating the integration of contraceptive services into other healthcare services.<br>• During the study time, 12 women requested PPIUD removal, with 10 desiring pregnancy and 2 due to husbands' disapproval.			
**Adaptable key components**<br>• The model of South-South cooperation can help expand postpartum IUD services in other regions.<br>• The paper highlights the importance of using a quality improvement approach to address barriers to PPIUD uptake, improve the quality of care, and collect and use data to monitor and evaluate program performance.<br>• Training by culturally similar providers.<br>• Immediate provision of equipment post-training.<br>• Use of data for advocacy.<br>• Proper data collection, regular reporting, and instrument and contraceptive stock-outs prevention are essential for the initiative's success.<br>• The example of Togo's success suggests adaptability to different contexts, which may serve as a model.			
**Results indicators**<br>• Service data include the number of women counseled, uptake of PPIUD services, and follow-up consultations.<br>• PPIUD expulsion rate as a measure of quality of care.			
**Use of digital tools**<br>• Digital tools such as smartphones or tablets were mentioned as potential aids in data collection and issue resolution, including early identification of PPIUD complications.<br>• The initiative encouraged all participating sites to report monthly PPIUD service delivery data from their standardized logbooks to their MOH, using these digital tools.<br>• Future research plans include determining how the health management and information system can track postpartum contraception uptake, potentially involving digital tools.			
**Level of gender responsiveness**<br>• Level 4 (Gender-specific). Targets specific needs of postpartum women for IUD services, addresses cultural barriers, and promotes informed contraceptive decision-making, but does not substantially challenge underlying gender norms or roles.			
Chukwumalu, K., et al. (2017) (9)	Somalia	Women of reproductive age who received post-abortion care services in four governmental health facilities in rural and urban districts in Puntland, Somalia. N=1,111.	This is an implementation research study that aimed to evaluate the effectiveness of a PAFP program in increasing contraceptive uptake among women seeking postabortion care (PAC) services. The study used a multipronged approach focusing on capacity building, assurance of supplies and infrastructure, and community collaboration and mobilization. An initial health facility assessment was conducted to identify areas for improvement. The study also involved the government in program design, implementation, reviews, and evaluation. The study used routine program data collected from four health facilities from January 2013 to December 2015.
**Strategies that report success**			

- To increase contraceptive uptake, 12 service providers received a three-week competency-based training for FP and PAC, with two staff receiving additional training to become master trainers themselves. The intervention included the provision of a range of contraceptive methods, counseling on contraceptive options, and follow-up visits to ensure the continuation of contraceptive use.
- The principal conclusions of the study were that a PAFP program can be effective in increasing contraceptive uptake among women seeking postabortion care services in Puntland, Somalia.
- The study also highlighted the importance of providing a range of contraceptive methods, counseling on contraceptive options, and follow-up visits to ensure the continuation of contraceptive use. When a greater variety of contraceptive choices were included, more women chose a LARC method.
- The paper acknowledges the need for reproductive health services, including gender-based violence and HIV, for internally displaced person (IDP) populations in humanitarian crises.
- The community engagement approach was successful in reaching the community with services and awareness raising on abortion. Enhanced acceptance of PAC in communities led to each facility employing two community health workers trained in conducting home visits and raising awareness in the community using specific educational and informational tools. Religious and community leaders became prominent supporters of PAC, focusing on community-driven strategies to facilitate women's access to health facilities for PAC services in a timely manner. Moreover, they regularly addressed the significance of PAC in community settings to help diminish any initial stigma surrounding these services. However, the study authors identified a gap in the community engagement approach, which did not specifically target men and youth. The paper also recognizes the influence of pronatalist religious and social conservatism in Somalia, which can elevate barriers to improving the availability and accessibility of quality PAC services.
- Establishing a training center was a crucial initiative within the program's capacity-building strategy, maintaining service providers' capacity for the project implementation.

Intervention results

- During the study period, of the 1,111 women who received PAC services, 98% received counseling for post-abortion contraception, of which 88% accepted a contraceptive method before leaving the facility; 30% of them chose a LARC method. The proportion of women accepting a contraceptive method increased from 16% in 2013 to 36% in 2015. When the contraceptive method mix increased over time, the percentage of women who opted for a LARC method increased from 10% (IUD) in the first six months to 30% (11% IUD and 19% implant) in the last six months; injectables decreased from 42% to 32% and the pill from 48% to 38%. The study also found that women younger than 25 were more likely to choose short-acting methods (none of them chose an IUD), while older women were more likely to choose LARC.

Adaptable key components

- Capacity building through competency-based clinical training, supportive supervision, and individual provider support.
- Establishment of a training center for service providers on FP, PAC, and other reproductive health training.
- Infrastructure improvement and supply chain management to ensure privacy, confidentiality, and uninterrupted services.
- Providing a range of contraceptive methods, counseling on contraceptive options, and follow-up visits to ensure the continuation of contraceptive use.
- Comprehensive monitoring and evaluation framework for data-driven programmatic decisions and performance optimization.
- Community collaboration and mobilization, especially with religious and community leaders, to clarify misconceptions about PAC and postabortion FP.
- Involvement of the MOH in all stages of the program cycle for good collaboration and successful program implementation.

Level of gender responsiveness

- Level 4 (Gender-specific). Increases contraceptive uptake in postabortion care and focuses on service provision and informed contraceptive decision-making without actively challenging prevailing gender norms.

Gbagbo, F. Y., et al. (2018) (10)	Ghana	Women attending health facilities for post-abortion care in Accra, capital of Ghana, including 90 private and 10 non-governmental organization facilities.	This interventional study employed a nested study design, originating from a larger study on decision-making for induced abortion. The providers were encouraged to capture details of women served in a monthly logbook and collected for analysis. The study was piloted using Marie Stopes, Ipas, and Ghana Health Service trained abortion providers with FP skills in 100 purposively selected health facilities mandated by law to provide safe abortion care services in Accra. The model focused on five key factors: contraceptive products, pricing, placement, promotion, and people. The effectiveness of the model was evaluated based on the increase in post-abortion contraception across the selected facilities.

Strategies that report success

- The implementation of the Post Abortion Contraception Model involved a two-week training, refresher training, and competency certification of service providers in abortion and FP services for four years.
- The model emphasized integrating products, pricing, placement, promotion, and people with options counseling before an induced abortion to improve post-abortion contraception uptake.
- The model is user-centered and focused on voluntary informed consent.
- The model was successful in rebranding PAC from "family planning" to "contraception" to increase acceptance, integrating PAC as a package for abortion care, and making PAC a major quality indicator of safe abortion.
- The model emphasized capacity building and technical updates for service providers to ensure quality and safe services.

Intervention results

- The model was found useful in significantly increasing PAC services and decreasing repeat induced abortions.
- The model led to a significant increase (90% average) in post-abortion contraception across the selected facilities following the intervention.

Adaptable key components

- Integration of products, pricing, placement, promotion, and people in the model led to an increase in PAC.
- The model was adaptable and successful in diverse settings, being piloted in 100 different health facilities.
- Early initiation of contraception following an abortion was found to reduce the risk of recurrent abortions.
- The model was successful with different groups of users, showing a significant decrease in repeat induced abortions.

Results indicators

- Voluntary and immediate PAC services provided.

Level of gender responsiveness

- Level 4 (Gender-specific). Focuses on increasing post-abortion contraception through a comprehensive model that relies on informed contraceptive decision-making but does not explicitly address gender relations or aim to transform them. While the study acknowledges the presence of partner pressure, it does not explicitly analyze how these factors might be impacting the program's effectiveness or propose specific strategies to address them.

Ingabire, R., et al. (2018) (11)	Rwanda	The study was conducted in Kigali, Rwanda, focusing on women who can use PPIUDs. The intervention was implemented in high-volume hospitals and health centers. The demand creation for PPIUD involved 32 participants in users focus groups, 14 health providers, 24 community health	This study evaluated a multi-level implementation science intervention to improve Rwanda's PPIUD services. The intervention included stakeholder engagement, training of healthcare providers, demand creation activities, and reimbursement for service delivery. The intervention involved training 49 in-clinic PPIUD counselors and 34 community health workers, to promote PPIUD to couples in-clinic and in the community. Two-day didactic counseling, insertion/removal, and follow-up trainings were provided to L&D and FP nurses. A PPIUD counseling flipchart was developed and delivered

		workers, and 150 women or couples attending ANC visits.	during ANC, labor and delivery, infant vaccination visits, or in the community. Data were collected by trained government clinic staff and managed by study staff. Stakeholders were involved from intervention development through dissemination of results. The intervention design also included the involvement of community health workers.

Strategies that report success

- The intervention included PPIUD counseling during ANC, labor and delivery, infant vaccination visits, or in the community. PPIUD counseling was effectively provided during ANC (20%), L&D (64%), postpartum (6%), and infant vaccination visits (10%). The intervention was designed with sustainability in mind, operating in government facilities led by trained government staff.
- The paper identified male involvement as a barrier to the uptake of PPIUDs. The intervention promoted PPIUDs to couples, acknowledging the role of male partners in FP decisions. The study found that most PPIUD removals were due to male partners not liking the method, highlighting the need for male involvement during counseling.
- Most women received counseling during L&D, which was found to be an effective time for counseling.
- The PPIUD service provision was limited to 6 weeks postpartum to coincide with the first infant vaccination visit, an opportunity to provide PPIUD counseling, service provision, or follow-up visitation.
- The intervention was designed with sustainability in mind, with the aim of operating in government facilities led by trained government staff.

Intervention results

- The intervention was associated with a significant increase in the uptake of PPIUD (defined in the study as up to 6 weeks post-delivery), from 7.7 per month before the intervention to 214.6 per month after the intervention; 62% of PPIUDs were inserted within 10 minutes of delivery of the placenta, 13% intra-cesarean, 17% between 10 minutes and 48 hours after delivery, and 8% between 4 and 6 weeks after.

Adaptable key components

- A multi-level intervention can effectively increase the uptake of PPIUD in low-resource settings and such interventions should be tailored to the local context and built on existing capacity and experiences.
- Early stakeholder involvement and collaboration can provide logistical and technical support, enhancing the effectiveness of the intervention.
- Performance-based financing incentives can offset administrative and time costs, potentially increasing the uptake of services requiring additional skills.
- Group counseling sessions can reach a larger number of women and increase awareness about PPIUDs.
- Using evidence-based counseling techniques could further improve the effectiveness of these strategies.
- The study highlights the importance of stakeholder engagement and demand-creation activities in promoting the uptake of FP services.

Use of digital tools

- Data were collected using tablets and a mobile data collection platform, Survey CTO v2.41, and uploaded into a Microsoft Access database for further analysis.

Level of gender responsiveness

- Level 4 (Gender-specific). Implements a multi-level intervention for PPIUD services involving male partners, but primarily focuses on increasing women’s contraceptive uptake without fundamentally changing gender dynamics.

Lori, J. R., et al. (2018) (12)	Ghana	Women in an urban district hospital in the Ashanti Region of Ghana. A total of 240 women were initially enrolled in the study, with 120 assigned to group ANC and 120 to standard, individual care.	The study employed a longitudinal, prospective cohort design with intervention and control groups. Women were assigned to either group ANC or standard, individual care at their first ANC visit. Data on FP use and breastfeeding were collected one-year postpartum using a structured survey. Statistical analyses were conducted using Pearson chi-square tests and logistic regression.

Strategies that report success

- The educational content of both group and individual ANC care was delivered through various methods, such as picture cards, role-play, storytelling, and teach-back. The group ANC model consisted of small groups of women with similar gestational age. Women in both the intervention and control group were encouraged to attend seven ANC visits.
- The study suggests that transmitting FP education in a comprehensible way during ANC increases interactions with health providers and has the potential for a long-term positive impact on contraceptive use.

Intervention results

- Group ANC participants had higher odds of using a modern FP method than those in individual care, even when controlling for factors like intention, age, gravida, religion, and education. Women who participated in group ANC were more likely to express an intention to use FP immediately postpartum and had significantly higher odds of using modern and non-modern contraception at one year postpartum compared to women in the individual ANC group (59% vs 19%, $p<.001$), and significantly higher odds of using a modern contraceptive method than the control group (AOR=8.063, $p<.001$).
- Group ANC led to a higher rate of exclusive breastfeeding for more than 6 months compared to individual care (76% vs. 50%, $p<.001$).

Adaptable key components

- Group ANC can be adapted to different contexts, showing higher uptake and continuation of postpartum FP compared to individual care.
- The facilitated discussion methodology in group ANC can be scaled up to larger groups.
- Group ANC can be a strategy to increase the uptake of postpartum FP in diverse settings.

Results indicators

- Use of any FP method at one year postpartum.
- Use of a modern method of FP at one year postpartum.
- Exclusive breastfeeding at 6 months postpartum.

Level of gender responsiveness

- Level 4 (Gender-specific). Increases FP use through group ANC, promoting women’s informed contraceptive decision-making, without directly challenging gender norms.

<table>
<tr><td>Torres, L. N., et al. (2018) (13)</td><td>United States</td><td>The study population consisted of women aged 18 to 45 who gave birth to a premature infant at a teaching hospital in Utah between April 2013 and January 2014. A total of 134 women were enrolled in the study, but the final analyses included 119 women who completed the 3-month follow-up questionnaire and had primary outcome data.</td><td>The study employed a single-blinded, one-to-one, randomized, controlled trial design. Participants were randomly assigned to either receive structured counseling with an emphasis on LARC (intervention group) or routine postpartum care (control group). The primary outcome was LARC use 3 months postpartum, and participants' contraceptive knowledge was assessed using applicable questions from a survey questionnaire. Statistical analyses used included chi-square tests and Student t-tests to detect differences in characteristics between the intervention and control groups, and one-way analysis of variance to compare the satisfaction of methods at follow-up.</td></tr>
<tr><td colspan="4">Strategies that report success
• The intervention group received structured counseling, following a script that included commonly available contraceptive methods and their risks, benefits, and side effects, explaining the most effective method first, with an emphasis on LARC by a FP specialist.
• The study supports the potential benefit of having an integrated in-hospital contraception consult service, similar to a lactation consult service, for contraceptive counseling in the immediate postpartum period.</td></tr>
<tr><td colspan="4">Intervention results
• Structured counseling on LARC increased participants' knowledge and use of LARC methods among women 3 months postpartum after a preterm birth compared to the control group.
• For every six women who received the counseling intervention, one additional woman was using a LARC method at 3 months. The intervention group had a significantly higher uptake of LARC methods at 3 months postpartum compared to the control group, which received routine postpartum care (51% vs. 31%, p<.05); they also had higher scores on contraceptive knowledge and were more likely to report being satisfied with their chosen contraceptive method.</td></tr>
<tr><td colspan="4">Adaptable key components
• Structured counseling: enhanced FP counseling immediately after a preterm birth significantly increased LARC method use at 3 months postpartum among women at risk of a subsequent preterm birth.
• In-hospital consultation: an integrated in-hospital contraception consult service, similar to a lactation consult service, can improve LARC use.
• Knowledge assessment: assessing contraceptive knowledge using applicable questions can help understand participants' confidence in their knowledge of LARC and non-LARC methods.
• Consideration of context: counseling strategies should consider the challenging circumstances of new mothers, such as dealing with adverse neonatal outcomes.</td></tr>
<tr><td colspan="4">Level of gender responsiveness
• Level 4 (Gender-specific). The intervention enhances knowledge and use of postpartum LARC methods, focusing on women's informed contraceptive decision-making, but without addressing underlying causes of gender roles or relations.</td></tr>
<tr><td>Wendot, S., et al. (2018) (14)</td><td>Kenya</td><td>Women at least 18 years old accessing safe abortion and post-abortion care services in nine private clinics in Western Kenya between November 2015 and July 2016. Interviews were completed with 769 women and 414 women completed a follow-up telephone interview.</td><td>The study was a pre-and post-intervention design. Baseline and post-intervention data were collected using in-person interviews on the day of the procedure, and follow-up telephone interviews to measure contraceptive uptake in the two weeks following abortion. The primary outcome was the proportion of women receiving any method of PAFP (excluding condoms) within those two weeks. Semi-structured interviews were also conducted with providers. Quantitative data were collected on socio-demographic status, counseling and provision of post-abortion FP, fertility intentions, previous contraceptive use and satisfaction with the service received. Chi-squared tests and multivariate logistic regression were used to determine significant differences between baseline and post-intervention, adjusting for potential confounding factors and clustering at the clinic level.</td></tr>
<tr><td colspan="4">Strategies that report success
• The intervention included a 1-day orientation that included balanced counseling, a counseling job-aide, and supportive supervision visits with health providers.
• Supportive supervision was identified as the providers' most effective intervention component.</td></tr>
<tr><td colspan="4">Intervention results
• The quality of counseling and satisfaction with services increased post-intervention.
• Increased same-day uptake of PAFP (aOR 1.94, p < 0.001) and LARC (aOR 1.72, p < 0.001) (but no overall increase in PAFP uptake two weeks post-abortion was observed).
• The proportion of women not taking an FP method on the day of the abortion due to lack of information declined from 11% to 4% (p=0.005) and due to undecidedness declined from 64% to 52% (p=0.013).
• The proportion of women reporting that the provider counseled them on ways to prevent pregnancy increased from 55% to 61% (p=0.051).</td></tr>
<tr><td colspan="4">Adaptable key components
• A quality management intervention involving a 1-day BCS orientation, a counseling job-aide, and supportive supervision visits was successful in improving PAFP counseling, provision, and satisfaction.
• Supportive supervision was identified as the most effective component of the intervention.
• The intervention was more effective when it targeted multiple aspects of performance.
• The intervention was effective in diverse settings, as it was implemented in multiple private clinics in Western Kenya.</td></tr>
<tr><td colspan="4">Level of gender responsiveness
• Level 4 (Gender-specific). Improves quality of post-abortion FP counseling and method uptake by using BCS (which promotes informed decision-making), targeting women's informed contraceptive decision-making without actively addressing gender power dynamics. While the study acknowledges the potential impact of gender dynamics (by asking for relationship status and partner support for contraceptive use), it does not explicitly analyze how these factors might be impacting women's decisions regarding PAFP.</td></tr>
<tr><td>Karra, M., et al. (2019) (15)</td><td>Sri Lanka</td><td>Women who delivered in six hospitals in Sri Lanka between September 2015 and January 2017. N=39,084. The intervention was part of FIGO's PPIUD Initiative launched in six countries: Nepal, Sri Lanka, India, Kenya, Tanzania, and Bangladesh.</td><td>The study was a cluster-randomized stepped-wedge trial conducted in six hospitals in Sri Lanka aimed at institutionalizing PPIUD as a routine component of ANC counseling and delivery. The intervention was administered to the first group of hospitals after a 3-month baseline period, while the second group received the intervention after 9 months of baseline data collection. The key treatment variable was a woman's exposure to the intervention at the time of her delivery, based on whether she delivered in a hospital after the start of the intervention. The study used an intent-to-treat (ITT) analysis to determine the impact of the intervention. The outcome</td></tr>
</table>

<table>
<tr><td></td><td></td><td></td><td>variables were binary, and the ITT effect was estimated using a simple linear regression.</td></tr>
<tr><td colspan="4">Strategies that report success
The intervention included several measures to enhance PPFP and PPIUD education and services: hosting educational workshops for healthcare personnel in maternity wards, such as doctors, midwives, nurses, and other hospital staff; offering PPFP counseling training for maternity care providers both in hospitals and MOH antenatal clinics; training doctors in the participating hospitals on how to insert PPIUDs; distributing PPFP informational leaflets in hospitals and MOH clinics for use during counseling sessions; showing a PPFP informational video in hospital waiting areas; supplying hospitals with the necessary tools for vaginal PPIUD insertion, specifically Kelley’s forceps, and copper-T IUDs; and carrying out ongoing monitoring and evaluation of the counseling and PPIUD insertion processes by SLCOG and FIGO.</td></tr>
<tr><td colspan="4">Intervention results
The intervention increased rates of counseling, from an average counseling rate of 12% prior to the intervention to an average rate of 51% after the rollout of the intervention (0.307; 95% CI 0.148–0.465).
The impact of the intervention on choice of PPIUD was less robust, with 4.1% of women choosing PPIUD prior to the intervention compared to 9.8% after the rollout of the intervention (0.027; 95% CI 0.000–0.054).
Women with at least higher secondary education or who lived less than 3 hours from the hospital were more likely to be counseled, reflecting either a provider bias or a refusal by certain women to be counseled.</td></tr>
<tr><td colspan="4">Adaptable key components
Incorporating PPIUD services into postpartum care is feasible and effective.
Factors such as providing leaflets during counseling, knowledge about PPIUD, opportunity to ask questions, and timing of counseling were identified as determinants of choice of PPIUD.
PPIUD is a convenient contraceptive option for women who cannot return for follow-up visits due to distance, travel costs, and time constrains.</td></tr>
<tr><td colspan="4">Results indicators
Percent of women given the opportunity to ask questions during counseling.
Percent of women who recalled benefits and/or disadvantages of PPIUD that trainers were expected to mention during counseling.
Percent of women counseled who gave consent to receive a PPIUD.</td></tr>
<tr><td colspan="4">Level of gender responsiveness
Level 4 (Gender-specific). Increases counseling rates and informed contraceptive decision-making but focuses on women’s health services without challenging underlying gender norms or how these norms might be impacting women’s experiences with the program or their choices regarding PPIUD.</td></tr>
<tr><td>Mossie, M. Y., et al. (2019) (16)</td><td>Ethiopia</td><td>The study was conducted in the Oromia region of Ethiopia in 2017-18, focusing on postpartum women and their interactions with the health system in districts without large scale FP programs. The sample included 103 health officials, health extension workers, and volunteers. The sample for in-depth interviews included two zonal and four district health officials, four heads of primary health care units, and 23 health extension workers from 18 health posts. Additionally, six focus group discussions were held with 70 volunteers referred to as “women development armies.”</td><td>The research employed a qualitative study design, using in-depth interviews and focus group discussions to assess the use, acceptability, and feasibility of tools for tracking PPFP counseling, women’s decision-making and PPFP use in Ethiopia’s community health system. It was part of a larger quasi-experimental study exploring how to maximize all contacts a woman has with the health system during pregnancy, childbirth, and the postpartum period. Community-level tracking tools included a modified Integrated Maternal and Child Health (IMCH) card with new PPFP content and a newly developed tool for pregnant and postpartum women. The study used a conceptual framework describing women’s decision-making and contraceptive use over the continuum of care.</td></tr>
<tr><td colspan="4">Strategies that report success
The modified IMCH card used in the intervention was found to be feasible and easy to use and complete by health extension workers for its ability to visually aid in education rather than relying solely on oral communication.
The modified card included prompts to facilitate tracking of PPFP choices and uptake over time, in addition to the standard documentation of ANC and child immunization and growth monitoring.
The women development armies used a new tool to promote referrals along the continuum of care that included PPFP use.</td></tr>
<tr><td colspan="4">Intervention results
Postpartum women in the intervention group were more likely to adopt PPFP than those in the control group.</td></tr>
<tr><td colspan="4">Adaptable key components
The modified IMCH card served to remind community health workers to conduct PPFP counseling during each ANC visit and to record the woman’s PPFP method choice, allowing health providers to follow up during childbirth and offer the method she chose or to use another method if she changes her mind.
The modified IMCH card helped community health workers to assess if postpartum women were at risk of pregnancy (based on months postpartum, breastfeeding practices, or the return of menses) and to counsel them about taking FP.</td></tr>
<tr><td colspan="4">Use of digital tools
The study mentions the potential of transforming paper-based records to digital health records to improve service integration and continuity of care.
The study suggests that digital platforms can incorporate nudges or prompts to strengthen continuity of care, track PPFP, and facilitate decision-making and timely adoption of contraceptives.</td></tr>
<tr><td colspan="4">Level of gender responsiveness
Level 4 (Gender-specific). The study primarily focuses on tracking PPFP counseling and informed contraceptive decision-making through the continuum of care. The study acknowledges couple or family discussion of PPFP as an input to uptake but does not analyze how gender dynamics or social norms might be impacting women’s experiences with PPFP or their decision-making processes.</td></tr>
<tr><td>Pradhan, E., et al. (2019) (17)</td><td>Nepal</td><td>The study was conducted in six large tertiary hospitals in Nepal. The population studied included all women who gave birth in these hospitals between September 2015 and March 2017. The sample size consisted of 75,587 women who consented to be interviewed while in</td><td>The study employed a stepped-wedge cluster randomized design. Hospitals were grouped into pairs based on geography and annual obstetric caseload, and then randomized into early or late intervention groups. The intervention introduced PPFP counseling in ANC and immediate PPIUD in two steps, with the first group of 3 hospitals starting 3 months after baseline data collection, and the second group starting 9 months after. The impact of the</td></tr>
</table>

<table>
<tr><td></td><td></td><td>the postnatal ward after delivery and before discharge from the hospital. The intervention was part of FIGO's PPIUD Initiative launched in six countries: Nepal, Sri Lanka, India, Kenya, Tanzania, and Bangladesh.</td><td>intervention was analyzed using a linear regression, controlling for hospital fixed effects and month fixed effects. An adherence-adjusted effect of the intervention on PPIUD uptake was calculated using an instrumental variable approach.</td></tr>
<tr><td colspan="4">Strategies that report success
• The intervention introduced PPFP counseling in ANC and immediate PPIUD.
• The intervention included informational workshops organized for female community health volunteers and general hospital staff; maternity care providers received training in postpartum family planning counseling, PPIUD insertion techniques, and how to manage complications; women were given PPFP leaflets during their counseling sessions; an information wall chart and educational video were set up in the hospital waiting area for patients to view; Kelley's forceps for vaginal PPIUD insertion were provided, along with IUDs supplied by the Ministry of Health and Population, free of charge at the point-of-care for women; each hospital designated one service provider as the facility coordinator for the program; and the program's counseling and insertion data were regularly reviewed by NESOG and FIGO.</td></tr>
<tr><td colspan="4">Intervention results
• Being exposed to the intervention increased PPIUD counseling among women by 25 percentage points [95% CI: 14–40 pp], and PPIUD uptake by four percentage points [95% CI: 3–6 pp].
• The adherence-adjusted estimate shows that, on average, being counseled due to the intervention increased PPIUD uptake by about 17 percentage points [95% CI: 14–40 pp].</td></tr>
<tr><td colspan="4">Adaptable key components
• Incorporating high quality PPIUD counseling and immediate IUD insertion into routine maternity care is feasible and can generate demand for PPIUD, reducing unmet need for contraception.</td></tr>
<tr><td colspan="4">Results indicators
• Percent of women reporting receiving counseling for PPIUD during ANC.
• Percent of women reporting receiving counseling for PPIUD after admission to the hospital for delivery.
• Percent of women given the opportunity to ask questions during counseling.
• Percent of women who recalled benefits and/or disadvantages of PPIUD that trainers were expected to mention during counseling.
• Percent of women who chose PPIUD and who had an IUD inserted postpartum.</td></tr>
<tr><td colspan="4">Level of gender responsiveness
• Level 4 (Gender-specific). Increases counseling and uptake of PPIUD, increasing women's contraceptive knowledge without explicitly analyzing how gender dynamics or social norms might be impacting women's experiences with the program or their choices regarding PPIUD.</td></tr>
<tr><td>Stephens, B., et al. (2019) (18)</td><td>Tanzania</td><td>Women who were receiving post-abortion care in 64 public health facilities in 4 districts in Dar es Salaam, Tanzania, from January 2016 to June 2018. Community mobilizers counseled 283,127 women on contraception and PAC, and 112,686 women were provided with 30-day follow-up visits.</td><td>Implementation research design that aimed to evaluate the effectiveness of expanding PAC coverage. Implementing key interventions included clinical training and mentorship for health providers (nurses, registered nurses/midwives, assistant medical officers, and medical/clinical officers) on PAC and PAFP; PAC service reorganization, equipment provision, and expanded method mix offering; standardization of PAC registers; and establishing community engagement to build awareness and referrals to PAC. Monitoring quality service indicators such as the providers' ability to appropriately assess the woman's reproductive health intentions, offer comprehensive contraceptive counseling, and document services on the woman's FP card. The study also tracked the number of women voluntarily adopting a contraceptive method.</td></tr>
<tr><td colspan="4">Strategies that report success
• Providers learned balanced counseling techniques to assess the woman's reproductive intentions and medical eligibility prior to methods counseling and then proceeded to review the methods starting with the most effective and progressing to the least effective and explain opportunities for method removal if the woman desired.
• Providers received no incentives for women adopting FP or specific methods.
• PAC service provision was not contingent upon women accepting FP.
• Community mobilizers received a 2-week training and orientation to identify, counsel, and provide respectful care to women using a systematic door-to-door approach.
• During home visits, community mobilizers sensitized women to the availability and safety of PAC using mobile-based job aids. The community mobilizers also worked to strengthen community awareness and access to health services. They provided counseling and referrals for contraceptives and PAC, and they also identified and referred women who needed PAC services.</td></tr>
<tr><td colspan="4">Intervention results
• Expanding PAC coverage to primary and secondary level facilities led to an increase in voluntary PAFP intake during a 30-month period: 80.6% (6,636/8,230), with a method mix of 58.3% implant, 18.9% intrauterine device, 13.7% pills, 8.6% injectables, and 0.5% permanent methods; 2,731 (40.9%) of those who adopted PAFP were ages 10–24 and had a comparable method mix.
• Mentored providers who counseled women on contraception and encouraged them on its timely initiation after abortion increased from 74.3% to 91.9%, those offering FP method information and supply prior to discharge increased from 88.6% to 94.1%, but the percentage of them who considered the woman's SRH intentions, including desire for future pregnancies, only increased from 91.4% to 92.6%.</td></tr>
<tr><td colspan="4">Adaptable key components
• Balanced counseling with no provider incentivization, reinforcing voluntarism and informed choice among women.
• Clinical training and follow-up mentorship for healthcare providers to improve their skills and knowledge retention.
• Reorganization of PAC services and provision of necessary equipment to ensure quality care.
• Offering an expanded mix of contraceptive methods to cater to the diverse needs of women.
• Standardization of PAC documentation tools for consistent data collection and monitoring.
• Establishing community linkages and referrals to ensure continuity of care and follow-up.
• Building capacities of facility managers through mentorship and supportive supervision.
• Regular data reviews and action planning for performance review and accountability within the health facility.
• Youth-friendly services that included offering the full FP method mix to adolescents and youth regardless of their age and marital or matriculation status.</td></tr>
<tr><td colspan="4">Results indicators
• Voluntary PAFP intake.
• Providers' ability to appropriately perform key contraceptive indicators:
  ○ Offer comprehensive contraceptive counseling (including opportunities for method removal if desired) and recommendation for the timely initiation of a method after abortion to protect from future unintended pregnancy or support appropriate spacing of pregnancies,
  ○ Assess the woman's SRH intentions (including desire for future pregnancy),</td></tr>
</table>

- Provide method information and supply,
- Document services on woman's FP card.

- Referrals for STI treatment.

Use of digital tools

- The investigators designed a tablet-based, open-sourced mobile application (CommCare) to guide mentorship visits.
- Mentors followed the mobile application through service steps and a quality checklist for service provision.
- The mobile application allowed to record provider performance during mentorship visits and calculate competency scores during service provision and over time.
- During home visits, community mobilizers sensitized women to the availability and safety of PAC using these tablets.

Level of gender responsiveness

- Level 4 (Gender-specific). Focuses on expanding post-abortion contraceptive coverage with BCS (which promotes informed contraceptive decision-making) and respectful care but does not actively seek to transform gender relations.

Haider, S., et al. (2020) (19)	United States	Women with infants aged 4.5 months or younger who were not using a LARC method and had not undergone sterilization in an urban academic medical center in Illinois. A total of 446 women were randomized for the study between January 2015 and January 2017. Women with infants aged 4.5 months or younger who were not using a LARC method and had not undergone sterilization were eligible to participate.	The research was a single site, randomized controlled trial conducted at an urban academic medical center serving a predominantly publicly insured patient population. The study aimed to evaluate the effectiveness of offering co-located contraceptive services with infant care to mothers after their well-baby visits in increasing the use of LARC at 5 months postpartum compared with usual care. Participants were randomly assigned to one of the two study arms. The primary outcome was the use of LARC at 5 months postpartum. Random assignment was implemented using the randomization module in the REDCap electronic data capture tool. Generalized linear models were used to estimate risk ratios.

Strategies that report success

- The study aimed to evaluate the effectiveness of offering co-located contraceptive services with infant care to mothers after their well-baby visits.

Intervention results

- Among women in the intervention group, only 17.8% accepted a co-located FP visit with infant care either the same day or scheduled with a future well-baby visit, although the concept was liked: 89.7% of those who accepted the intervention and 69.7% of those who declined it liked the intervention. Insufficient time to stay for the visit was the biggest barrier to uptake (21.6% of those who declined). Future research should test a version of this intervention designed to overcome barriers that participants reported.
- Among women who accepted the contraceptive visit, satisfaction ratings were very high, with 100% of participants reporting that they were very satisfied with the counseling they received. Additionally, 96.7% of participants reported feeling very comfortable with birth control at the visit, 90.0% that the time spent on counseling was right, and 86.7% reported that the visit was very convenient. When asked if they would recommend a linked contraceptive visit with well-baby care to a friend, 64.2% of women chose definitely would and 17.9% chose probably would.
- Women who accepted the visit were more likely to use a LARC method at 5 months than women in the control group (RR 1.97; 95% CI, 1.26–3.07).

Adaptable key components

- The concept of co-located contraceptive care at well-baby visits was well-received by mothers.

Results indicators

- The likability and satisfaction of co-located PPFP services with infant care was measured with these indicators:
  - Liked the intervention (a lot, a little, not much, not at all).
  - What was liked (missed postpartum follow up, not planning on attending postpartum follow up, in need of birth control, or convenient, other, none).
  - What was not liked (no time to stay longer, too tired after baby's visit, uncomfortable seeing new provider, children were there, worried about insurance, other, none).
  - Chose to use these services again (definitely, probably, probably not, definitely not).
  - Reasons for declining the intervention: (no time to stay longer, too tired to stay longer, uncomfortable seeing new provider, children were present, worried about insurance coverage, do not want birth control, already have birth control, already have a scheduled postpartum visit, other, none).
  - Satisfaction with counseling (very satisfied, somewhat satisfied, not very satisfied, not at all satisfied).
  - Comfortable with birth control at this visit: very comfortable, somewhat comfortable, somewhat uncomfortable, very uncomfortable).
  - Convenience of visit (very convenient, somewhat convenient, neither convenient nor inconvenient, somewhat inconvenient, very inconvenient).
  - Time spent on counseling (too little time, just the right amount of time, a little too much time, way too much time).
  - Received new birth control method today (yes, no).
  - Recommend to a friend (definitely, probably, probably not, definitely not).
  - Rating of quality of care (excellent, very good, good, fair, poor).

Use of digital tools

- The study used REDCap, a secure, web-based application, for data collection and management.

Level of gender responsiveness

- Level 4 (Gender-specific). Offers co-located contraceptive services with infant care to enhance informed contraceptive decision-making. Considers marital status or living arrangements, acknowledging the potential impact of gender dynamics, but does not develop actions to change gender norms or relations.

Huber-Krum, S., et al. (2020) (20)	Nepal	Women in Nepal who had recently given birth between 2015 and 2017 in two hospital clusters comprised of 6 hospitals. N= 75,893. The study was part of the FIGO's PPIUD Initiative launched in six countries: Nepal, Sri Lanka, India, Kenya, Tanzania, and Bangladesh.	The study utilized a cluster, stepped-wedge design to randomly assign two hospital clusters to begin the intervention at different times. The intervention involved training health volunteers and hospital staff on PPFP counseling and training doctors and nurses who provided labor and delivery services on PPIUD insertion. They provided counseling aids and informational tools and designating a facility coordinator in each hospital. Surveys were conducted after delivery but before discharge, and then at one year and two years postpartum. The intent-to-treat effect of the intervention was estimated using weighted, linear probability models and the adherence-adjusted effect was estimated using an instrumental variable approach.

Strategies that report success

- Counselors provided information about all FP available methods, placing emphasis on the advantages of PPIUD. PPIUD services, which were only available at limited hospitals in the country, were provided free of charge, including the device, insertion, and removal.

<table>
<tr><td colspan="4">Intervention results
• Pregnant women who received counseling in the ANC period had the option to provide advance consent to PPIUD insertion, and their medical charts were marked with their stated decision. These women were consented again at the time of delivery to confirm their choice for PPIUD insertion. However, only 42.8% of women were counseled during the intervention period at one year (vs 10% in the control group) and 42.7% at two years (vs 9.9% in the control group), suggesting lack of compliance to the protocol. Expanding coverage may require addressing barriers such as hospital management issues, limited supplies, and human resource constraints, and providing additional support.
• The effect of the intervention on the probability of PPIUD use was 12.0 pp [95% CI: 6.1, 16.4 pp] at year one and 10.5 pp [95% CI: 4.7, 15.8 pp] at year two. However, the intervention only increased use of modern contraceptives by 3.8 percentage points [95% CI: 0.1, 9.5] at one-year postpartum and only 0.3 percentage points [95% CI: 3.7, 4.1] at two years.</td></tr>
<tr><td colspan="4">Adaptable key components
• Training providers to counsel women during ANC and in early L&D, but not during acting labor.
• Integrating ANC counseling and IUD insertion services can significantly increase the uptake of PPIUDs and other modern PPFP, but the integration may be challenging in settings where it is not the norm.
• For ANC counseling to increase postpartum contraceptive use, counseling may need to be provided in a wider range of prenatal care settings and at multiple time points.
• Healthcare providers should be trained on contraceptive counseling and PPIUD insertion, with the goal of expanding the available method mix and meeting postpartum women's contraceptive needs.</td></tr>
<tr><td colspan="4">Use of digital tools
• Counseling aids and informational tools, including leaflets, wall charts, and videos, were provided and distributed during counseling and displayed in hospital waiting areas.</td></tr>
<tr><td colspan="4">Level of gender responsiveness
• Level 4 (Gender-specific). Focuses on training providers in counseling and PPIUD insertion, promoting informed contraceptive decision-making. Acknowledges gender norms and social pressure to have children but does not analyze how these might be impacting women's experiences with the program or their decision-making processes.</td></tr>
<tr><td>Lacy, M. M., et al. (2020) (21)</td><td>United States</td><td>Women in Tennessee insured under Tennessee Medicaid programs (TennCare) who were eligible and desired immediate postpartum or post-abortion LARC. Eligibility was based on the American College of Obstetricians and Gynecologists (ACOG) recommendations. Six hospital sites participated in the project from March 2018 to March 2019.</td><td>The study was a statewide quality improvement project based on the Institute of Health Improvement Breakout Collaborative model. An evidence-based IPP LARC toolkit was created to provide guidance to the sites, and monthly huddles occurred during the year of implementation. The project used a web-based data entry system through REDCap software for data collection and analysis. The project also involved the development of LARC specific patient consent forms and the establishment of coding, billing, and reimbursement procedures for LARC devices. A team of champions was identified at each institution to record patient education and input all data related to the project into REDCap software. The primary outcome measure was to determine whether the participating institution was providing the option of IPP LARC, and the secondary outcome measure was the percent of LARC devices placed for each type provided by the institution.</td></tr>
<tr><td colspan="4">Strategies that report success
• Steps to guard against coercion while ensuring proper counseling, woman's choice, and access were stressed when implementing IPP LARC as a contraceptive option. However, most teams found difficult to measure the number of women desiring IPP.
• Doctors and nurses were trained on the ethical considerations, benefits, and risks of IPP placement of LARC devices to provide patient-centered comprehensive contraception counseling and were reminded that IUD removal or replacement should be completed whenever a woman had such desire.
• Contraception education materials for users were published in English and Spanish to increase accessibility.</td></tr>
<tr><td colspan="4">Intervention results
• The project successfully increased access to IPP LARC through woman-centered counseling and ensuring reimbursement for devices: the number of LARC devices placed by 5 teams across the state (all but one team) in one year increased from 112 (43 IUDs and 69 implants) to 2,012 (776 IUDs and 1,236 implants).</td></tr>
<tr><td colspan="4">Adaptable key components
• Implementation of an evidence-based toolkit to provide guidance to sites.
• Regular meetings or "huddles" to discuss progress and address challenges, a strategy that can be scaled up .
• Identification of a team of champions at each institution to lead the project.
• Development of specific patient consent forms for the implementation of new procedures.
• Use of a web-based data entry system for data collection and analysis.
• Provider and nurse education on ethical considerations, benefits, and risks of procedures.</td></tr>
<tr><td colspan="4">Use of digital tools
• The study utilized REDCap software for data input related to the project at each institution.</td></tr>
<tr><td colspan="4">Level of gender responsiveness
• Level 4 (Gender-specific). Increases access to IPP LARC, focusing on women's health needs and informed contraceptive decision-making but does not analyze how gender dynamics or social norms might be impacting women's experiences with IPP LARC or their decision-making processes.</td></tr>
<tr><td>Pearson, E., et al. (2020) (22)</td><td>Tanzania</td><td>Postpartum Tanzanian women aged 18 or older who delivered in one of five study hospitals between January and September 2016. N=15,264. The intervention was part of FIGO's PPIUD Initiative launched in six countries: Nepal, Sri Lanka, India, Kenya, Tanzania, and Bangladesh.</td><td>The research was a cluster-randomized controlled trial that evaluated an intervention sought to improve PPFP and that included counseling on PPFP during ANC and delivery care and immediate PPIUD insertion. The intervention was introduced in a staggered manner across different facilities, a design known as a stepped-wedge trial. The study used a difference-in-differences approach to compare outcomes between the pre-and post-intervention period in the treatment and control group. The study also conducted an intervention adherence-adjusted analysis using an instrumental variables estimation. The study controlled for hospital and month fixed effects and included women's socio-demographic characteristics in the adjusted model. The study used linear probability models to estimate the effect of the intervention in percentage points.</td></tr>
<tr><td colspan="4">Strategies that report success
• Among women counseled, determinants of choosing PPIUD included receiving an informational leaflet during counseling and being counseled after admission for delivery services.</td></tr>
</table>

Intervention results

- The intervention increased PPIUD counseling by 19.8 percentage points (95% CI: 9.1 – 22.6 pp) and choice of PPIUD by 6.3 pp (95% CI: 2.3 – 8.0 pp). Although counseling increased post-intervention in Group 1 hospitals from less than 4% up to 22% and 40%, respectively, in Group 2 hospitals there was no increase. If all women had been counseled, there would have been a 31.6 pp increase in choice of PPIUD (95% CI: 24.3 – 35.8 pp).

Adaptable key components

- Increasing coverage and incorporating quality PPFP counseling during ANC and delivery care can improve women's access to PPFP services, including PPIUD.
- PPIUD intake can be increased when:
  - Introducing PPIUD insertion as an integrated part of delivery services.
  - Providing counseling and informational materials on PPIUD and counseling after admission for delivery.
  - Ensuring universal PPIUD counseling.

Results indicators

- Percent of women reporting receiving counseling for PPIUD during ANC.
- Percent of women reporting receiving counseling for PPIUD after admission to the hospital for delivery.
- Percent of women given the opportunity to ask questions during counseling.
- Percent of women who recalled benefits and/or disadvantages of PPIUD that trainers were expected to mention during counseling.
- PPIUD uptake was measured as a dichotomous variable based on either the woman's or the provider's report of the insertion.

Use of digital tools

- The study utilized the CommCare data collection application for gathering responses from participants.

Level of gender responsiveness

- Level 4 (Gender-specific). Increases counseling and choice of PPIUD, targeting women's informed contraceptive decision-making. Considers marital status or living arrangements, acknowledging the potential impact of gender dynamics, but does not develop actions to change gender norms or relations.

Reyes-Lacalle, A., L., et al. (2020) (23)	Spain	Pregnant women receiving prenatal care at twenty public Primary Care facilities in Catalonia, between October 2015 and March 2016. 1,004 women were recruited and 975 enrolled.	The study was a community-randomized trial conducted in Catalonia that evaluated the effectiveness of a perinatal contraceptive counseling intervention. The research team presented the study to the participating midwives and other health staff at each reproductive and sexual health care unit before implementation. At half of the centers, midwives provided standard perinatal contraceptive counseling (control group). At the other half, the intervention consisted of supplemental contraceptive counseling at different time points during pregnancy and postpartum, including a leaflet and a blog with information about all contraceptive options, a short reminder message in the mobile phone during the third quarter of pregnancy, and a face-to-face or a virtual meeting lasting 20 min at 2 weeks postpartum. Midwives used ad hoc questionnaires to collect information at week 30 of pregnancy (recruitment), and week 6, month 6 and month 12 postpartum. Data were collected using the electronic clinical record program (ECAP) and analyzed using SPSS v. 23.0.

Strategies that report success

- A perinatal contraceptive counseling intervention that includes a face-to-face session and the provision of printed or electronic materials can significantly increase contraceptive use and decrease the use of low-effectiveness methods.

Intervention results

- At month 12, more women in the intervention group, particularly those originally from Spain (77% of the sample), used more effective contraception and less women used less effective contraceptives vs. those of the control group ($p = 0.006$). On multivariate analysis, the additional counseling on Spanish women resulted in a higher use of highly effective methods (OR 0.66 [CI 95% 0.46–0.95], $p = 0.025$).
- Contraceptive counseling had a scarce effect on migrant women and women with low literacy, who had a higher use of effective and very effective contraceptives even when they were in the control group. This may be explained by an observed bias in standard clinical practice towards fostering use of very effective contraceptives among women with low education.
- Satisfaction with counseling and the type of contraception chosen was higher in the intervention group: among these, 97.2% were very satisfied or satisfied with the counseling received and 82.4% with the contraceptive chosen vs. 88.7% ($p < 0.0001$) and 74.5% ($p = 0.003$), respectively, of women in the control group. No relationship was found between satisfaction with the counseling intervention and the use of effective or very effective methods ($p = 0.472$).

Adaptable key components

- Supplemental contraceptive counseling at different time points during pregnancy and postpartum is effective in increasing contraceptive use and satisfaction with the method chosen.
- The classification of contraceptives as very effective (sterilization, LARC such as copper IUD, levonorgestrel intrauterine systems, and implants), effective (injectables, contraceptive patches, pills, and vaginal rings), and somewhat effective (diaphragms, withdrawal, sponges, female and male condoms, fertility awareness-based methods, and spermicidals) can be used to assess the effectiveness of contraceptive use.

Results indicators

- A 5-level scale, ranging from very unsatisfied to very satisfied, to assess satisfaction with the contraceptive counseling intervention and the method chosen.

Use of digital tools

- Digital tools were used in the form of electronic clinical record program (ECAP) for data collection.
- The intervention included the provision of contraceptive information through printed or electronic materials.
- At week 37 of pregnancy, the women received a SMS reminder to consult contraception information provided in the blog.
- Remote interviews were conducted via telephone or videoconference by Skype when face-to-face interviews were not possible.

Level of gender responsiveness

- Level 4 (Gender-specific). Enhances contraceptive use through additional counseling, focusing on women's informed contraceptive decision-making. Considers the partner's involvement and the marital or civil union status and acknowledges the study limitation that intimate partner violence was not analyzed. However, it does not analyze how gender dynamics or social norms might be impacting women's experiences with contraception or their decision-making processes.

Sitrin, D., et al. (2020) (24)	Ethiopia	Pregnant women in two adjacent districts in the Oromia Region, Ethiopia, with 8 health centers and 47 health posts. 772 women were enrolled and interviewed through community outreach between February and March 2017 and re-interviewed in May 2018.	The study used a quasi-experimental design with a controlled trial approach to estimate the additional effect of systematically integrating PPFP into community-level services for pregnant and postpartum women in a setting where home childbirth is common. Primary health care units were randomly assigned to intervention and comparison arms to reduce selection bias. Before randomization, all health centers received training on PPFP counseling for ANC, labor and delivery, and postnatal providers; PPIUD insertion training for labor and delivery providers; insertion kits and registers; and post-training. Health extension workers in the intervention arm received additional PPFP counseling; a refresher on implant insertion; quarterly supervision; modified integrated MCH cards with space to document PPFP counseling, method choice, and method adoption at each contact (ANC, PNC, immunization, and growth monitoring); modified cards for pregnant women that included contraceptive method choice; and were tasked with training volunteer women development armies on PPFP counseling, tracking choices and uptake, pregnancy risk, and referrals for FP. Data were collected through supervision of health centers and intervention health posts, and service statistics were extracted from records kept at centers and posts.
**Strategies that report success**<br>• Integrating PPFP into community-level services for pregnant and postpartum women and infants may have additional benefits in addition to PPFP services provided at health facilities.			
**Intervention results**<br>• Integrating PPFP into community-level services increased the adoption of modern contraception among women who delivered at home, who had a higher PPFP adoption by one year postpartum in the intervention arm (35.2%, 95% CI: 28.8–42.4%) versus comparison arm (27.8%, 95% CI: 22.2–34.4%). In the adjusted Cox regression model, women who delivered at home in the intervention arm were 45% more likely to adopt contraception (adjHR1.45, CI: 1.01–2.07). The intervention did not show a significant difference in contraceptive adoption for women who delivered in a facility.<br>• Contacts with health extension workers at health posts and immunization outreach contributed more to the success of the intervention than home visits, probably due to the shorter time spent on home visits compared to visits at health posts.			
**Adaptable key components**<br>• Integration of PPFP into community-level services, especially in contexts where home childbirth is common, can improve contraceptive use and may have additional benefits for pregnant and postpartum women and infants.<br>• The use of a pictorial tool to share information on PPFP and track women's PPFP choices and uptake can be effective.<br>• The community intervention can contribute to improved performance at health centers, possibly due to increased awareness and conversations on PPFP.			
**Results indicators**<br>• Time from delivery to uptake of modern contraception.			
**Level of gender responsiveness**<br>• Level 4 (Gender-specific). Integrates PPFP into community-level services, focusing on women's contraceptive informed contraceptive decision-making and use. Considers marital status but does not analyze how gender dynamics or social norms might be impacting women's experiences with the program or their decision-making processes.			
Wu, W. J., et al. (2020) (25)	Nepal	Recent postpartum women from seven village wards in a single municipality in rural Nepal. In the quantitative component, there were 445 women in the pre-intervention group and 508 in the post-intervention group. In the qualitative, there were 3 health providers, 4 women enrolled in the program, and 3 husbands who attended contraceptive counseling.	The study was a pre-post-intervention evaluation conducted in rural Nepal. The primary outcome was modern contraceptive use among recently postpartum women, measured pre-and one-year post-intervention. The study used both quantitative and qualitative methods. The study stratified analysis by early postpartum and late postpartum periods. Bivariate analyses were performed to examine characteristics that differed significantly between the pre- and post-intervention groups. The study used the Population Council's Balanced Counseling Strategy toolkit, adapted for community-based, CHW-delivered, postpartum contraceptive counseling.
**Strategies that report success**<br>• The intervention involved CHWs aided by mobile technology delivering patient-centered, home-based ANC and PNC counseling.<br>• The intervention contributed to increasing contraceptive use through knowledge transfer (which extended to husbands and mothers in law), demand generation, referrals to healthcare facilities, and follow-up.			
**Intervention results**<br>• Modern contraceptive use increased from 29% pre-intervention to 46% post-intervention (p < 0.0001). Adjusting for age, caste, and household expenditure, time since delivery, and sex of child in the index pregnancy, postpartum women one-year post-intervention had more than twice the odds (OR 2.3; CI 1.7, 3.1; p < 0.0001) of using a modern contraceptive method as compared to pre-intervention. Among non-users in the post-intervention group, 72% stated it was because their "husbands were away."<br>• In a stratified sub-analysis of modern contraceptive use by time since delivery, early postpartum women in the pre-intervention group had a 44% use rate as compared to 64% in the post-intervention group (p < 0.0001). The increase was mostly due to LAM, which increased from 34% to 56% (p < 0.0001). For women in the late postpartum period, modern contraceptive use increased from 16% preintervention to 30% post-intervention (p =0.0002). Increase in injectable use accounted for most of the change, as it increased from 6% pre-intervention to 21% (p < 0.0001) post-intervention.<br>• The contraceptive counseling was well-received by participants, and the visual aids proved useful. Some preferring counseling at home rather than at the health facility.<br>• All women and husbands interviewed perceived the counseling as non-coercive and appreciated the information provided.<br>• However, none of the women interviewed viewed the decision-making process regarding FP as exclusively their own and mentioned that the opinions of spouses and family members were crucial in determining women's contraceptive choices. Although greater knowledge among women facilitated their decision-making, the prevalent lack of personal agency meant that many women needed their husbands' and family members' approval. Efforts by CHWs to include husbands in counseling were often unsuccessful due to their absence. The limited agency of women was also evident in their reluctance to provide detailed suggestions for improving the current situation, with most recommendations coming from providers and husbands instead. Future studies are essential to identify effective methods for promoting shared decision-making in this context, supporting women in realizing their reproductive objectives.			
**Adaptable key components**<br>• A community-based, patient-centered contraceptive counseling intervention supported by mobile technology and integrated into longitudinal care delivered by CHWs was an effective strategy for improving the uptake of modern contraception among postpartum women in a rural area.<br>• Implementation of the Balanced Counseling Strategy toolkit, adapted for community-based, postpartum contraceptive counseling.<br>• Inclusion of visual aids to improve understanding during counseling sessions.<br>• Regular follow-ups by CHWs and relationship building with users through longitudinal care models.<br>• Facilitation of patient autonomy in decision-making by providing comprehensive information about contraceptive methods.			

<table>
<tr><td colspan="4">• Integration of the intervention into a broader RMNCH program.</td></tr>
<tr><td colspan="4">Results indicators
• Modern contraceptive use among recently postpartum women.</td></tr>
<tr><td colspan="4">Use of digital tools
• The study used CommCare, an open-source platform, to build a customized mobile application for data collection and displaying counseling content, with an additional module for the BCS. The study engineer trained CHWs on CommCare use and data collection.
• The CHWs used the mobile application to individualize the counseling based on a woman's responses to the BCS questions, based on which the application provided instructions for the CHW to set aside less relevant contraceptive options and individualize the counseling. The CHW recorded the woman's chosen method in the mobile application. At subsequent visits, the CHW collected information on initiation, barriers to access, and continuation.
• The use of visual aids during counseling sessions improved understanding and helped deliver patient-centered, home-based ANC and PNC counseling, that proved to be a useful tool for supporting their counseling efforts.</td></tr>
<tr><td colspan="4">Level of gender responsiveness
• Level 4 (Gender-specific). Increases contraceptive use through BCS (which promotes informed contraceptive decision-making), community-based counseling, targeting women's needs without explicitly addressing gender dynamics.</td></tr>
<tr><td>Espey, J., et al. (2021) (26)</td><td>Rwanda</td><td>Postpartum women who received postpartum FP educational counseling and delivered in one of six public health facilities in Kigali, Rwanda, from August 2017 to October 2018. 12,068 women received PPFP educational counselling and delivered at a study facility.</td><td>Multilevel intervention designed to increase integration and supply and demand for PPFP services during ANC, L&D, and infant vaccination services. The study collected demographic data during the service delivery intervention, only for postpartum implant and IUD users. The study involved the development of an educational flipchart on PPIUD for use in group and one-on-one counseling. The study analyzed the relationship between the number of promotions and the uptake of LARC methods.</td></tr>
<tr><td colspan="4">Strategies that report success
• A multilevel intervention was developed to increase the supply, demand, and sustainability of postpartum LARC methods. The intervention included promotional counseling on PPIUD for users, insertion and removal PPIUD training for providers, refresher training on implant insertion and removal, and involvement of MOH stakeholders.
• The intervention was well-received by both users and providers.
• Postpartum implant users reported no adverse reactions or discomfort, and no user requested a removal up to the 6-week postpartum infant vaccination visit.</td></tr>
<tr><td colspan="4">Intervention results
• Most participants received only one promotion (89%), while 9% received two, and 1% received three or four promotions. The average number of promotions per user was the same between the two LARC methods. Partners or fathers were included in the promotions if they were present.
• PP implant and PPIUD uptake significantly increased after the implementation of the intervention, from 30 PP implants and 8 PPIUD per month to 84 and 225 per month, respectively; however, 61.7% declined a PP LARC method.
• Receiving more promotions was associated with uptake for PP implants (test for trend, X2=65.8, p<0.0001) and PPIUDs (test for trend, X2=26.9, p<0.0001). Repeat promotions increased the opportunity for male involvement.
• Providers reported high ease of LARC insertion, and users reported minimal insertion average anxiety or pain: 2.1/10 (SD=2.6) anxiety and 2.0 (SD=2.6) pain for implants and 1.7/10 (SD=0.9) anxiety and 1.8 (SD=1.0) pain for IUDs.</td></tr>
<tr><td colspan="4">Adaptable key components
• Multilevel interventions that increase supply and demand for services based on promotional counseling on PPIUD for users, insertion and removal PPIUD training for providers, refresher training on implant insertion and removal, and involvement of MOH stakeholders.
• Promotional counseling for users and training for providers are crucial components of successful strategies.
• Involving key stakeholders, such as the Ministry of Health, is essential for the success and sustainability of interventions.
• Repeat promotions and male engagement in discussions can improve the uptake of services.
• Integrating services into routine care within ANC, L&D, and infant vaccination services can be a successful strategy in different contexts.</td></tr>
<tr><td colspan="4">Results indicators
• Number of participants who received the intervention (at least one PPFP promotion) and delivered at a study health facility.
• Number of participants declining a PPFP method.
• Postpartum implant and IUD users' anxiety Likert scale (1 = no anxiety or pain to 10 = maximum anxiety or pain).</td></tr>
<tr><td colspan="4">Level of gender responsiveness
• Level 4 (Gender-specific). Implements a multilevel intervention to increase uptake of postpartum LARC methods, promoting informed contraceptive decision-making and male engagement but without seeking to transform gender norms and relations.</td></tr>
<tr><td>Karra, M., et al. (2022) (27)</td><td>Malawi</td><td>Married women aged 18 to 35 in Lilongwe, Malawi who were either pregnant or had recently given birth between September 2016 and January 2017. 2,143 women completed the baseline survey and were randomized into the intervention or control groups.</td><td>Nonblinded, randomized, controlled trial conducted to assess the causal impact of improved FP access on contraceptive use and pregnancy spacing. Participants were stratified based on various factors and then randomized into treatment or control groups. The intervention group received, over 2 years, FP counseling, free transportation to an FP clinic, free FP services, and treatment for contraceptive-related side effects. The study conducted an intention-to-treat analysis on contraceptive use and pregnancy since the index birth, measured at the 2-year follow-up. Both unadjusted and adjusted analyses were performed, with the latter including various woman-level and neighborhood-level controls.</td></tr>
<tr><td colspan="4">Strategies that report success
• Increased access to FP has a positive impact on women's contraceptive uptake.</td></tr>
<tr><td colspan="4">Intervention results
• The study's results demonstrate strong and significant effects from expanding access to FP. The intervention led to a 5.9 percentage point increase [95% CI: 2.4, 9.4] in contraceptive use after 2 years of exposure to the intervention, primarily through increased use of contraceptive implants.
• The intervention resulted in longer birth intervals among women in the intervention group compared to the control group:
  ○ The probability of becoming pregnant with 2 years after the index birth was reduced by 3.7 pp [95% CI: −6.2, −1.1].</td></tr>
</table>

- The hazard of pregnancy was 43.5% lower 2 years after the index birth in the intervention group, based on a hazard rate of 0.565 [95% CI: 0.387, 0.824].

Adaptable key components

- Providing FP counseling sessions to educate women about contraceptive use and birth spacing.
- Offering free transportation to FP clinics to improve access to services.
- Ensuring free PF services or financial reimbursement for services obtained elsewhere to reduce financial barriers.
- Offering treatment for contraceptive-related side effects to address health concerns.
- Tailoring interventions based on the local context and barriers to FP, such as lack of knowledge, access, and fear of side effects.
- The intervention design was informed by a literature review of key barriers to FP, preliminary assessments, and discussions with key stakeholders.

Level of gender responsiveness

- Level 4 (Gender-specific). Increases contraceptive use and birth spacing, targeting women informed contraceptive decision-making. As acknowledged in the study limitations, while the study provided guidance to women on ways to involve their husband or male partner via counseling and dialogue, leaving up to the women how much she wanted to involve him in any part of the counseling or intervention, it did not specifically address the social norms concerning community or partner perspectives on FP, alleging that it would have required a larger, cluster randomized, multiarm trial.

Ijarotimi, O., et al. (2023) (28)	Nigeria	Postpartum women attending infant vaccination clinics in a Nigerian tertiary hospital in Ile-Ife. For the qualitative aspect, 12 participants were interviewed. For the quantitative aspect, 715 women participated.	Operations research study conducted in a Nigerian tertiary hospital and a pilot study conducted at three FP and vaccination sites to test an intervention integrating FP education with infant vaccination visits. Formative assessment was carried out using users records and key-informant interviews. Pre-and post-integration questionnaires were administered to the women attending the infant vaccination clinics. The qualitative data were analyzed using NVivo version 12 and the quantitative data using Stata version 17. The study also involved training of infant vaccination clinic staff in FP education.

Strategies that report success

- The integration of FP education and infant vaccination services is a feasible and acceptable strategy for increasing contraceptive use among postpartum women, as vaccination clinic staff were willing to take on FP education along with their current duties—although inadequate training and time constraint were identified as key barriers.

Intervention results

- The integration of FP education with infant vaccination visits was associated with significant increases in the knowledge of contraception (25.7% vs 34.7%, p=0.001), intention to use contraception (31.2% vs 38.2%, p=0.001), and number of new acceptors of FP (487 vs 664, p=0.001).
- The knowledge of side effects and mechanisms of action of contraceptives significantly increased after they were provided additional FP education by trained FP providers in the vaccination clinics from 25.7% to 34.7% (p=0.001) for side effects and from 9.7% to 14.5% (p=0.003) to mechanisms of action.

Adaptable key components

- Integration of targeted FP education and referral with infant vaccination services to increase contraceptive use among postpartum women.
- Regular training and retraining of FP providers to update their knowledge of quality and evidence-based contraceptive information and guidelines.

Results indicators

- How soon women who desire to have another child want to get pregnant again (within 24 months or after 24 months).
- Desire to get pregnant again or have another baby (yes/no).
- Desire for contraception to delay/space pregnancy amongst non-current contraceptive users (yes/no).
- Preferred contraceptive of non-current contraceptive users who desire contraception (condoms, IUD, implant, injectable, pills, tubal ligation).

Level of gender responsiveness

- Level 4 (Gender-specific). Integrates FP education with infant vaccination, targeting women's informed contraceptive decision-making. Considers the marital status without addressing the underlying gender dynamics that might play a role in contraceptive choices.

Level 3 of gender responsiveness: gender-sensitive (N=2)

Melnick, A. L., et al. (2016) (29)	United States	Vulnerable, low-income, first-time pregnant, at or before 33 weeks' gestation, at risk for unintended and short-interval pregnancies at five sites of three Nurse-Family Partnership programs in Washington State. N=337.	Randomized controlled trial design to examine the effectiveness of home-based hormonal contraceptive dispensing for women at risk of unintended pregnancy. Participants were randomly assigned to either the enhanced care (intervention) group or the usual care (control) group using block randomization. Nurses visited participants on a weekly or biweekly basis and provided education, counseling, and referrals to community primary care and FP clinics. The intervention group received the additional opportunity to receive no-cost, home-dispensed hormonal contraceptives, while the control group received standard home visit services. Longitudinal Poisson mixed-effects regression analysis and survival analysis were used to examine group differences in contraceptive use and time to subsequent pregnancies. The study aimed to enhance the existing NFP program and evaluate the impact of the intervention on postpartum contraceptive use and subsequent pregnancies

Strategies that report success

- The postpartum provision of hormonal contraceptives at home in the context of the Nurse-Family Partnership home visit program is an intervention that addresses barriers to contraceptive access by supporting nurses' dispensing and administering of hormonal contraceptives during home visits with women at risk for unintended and short-interval pregnancies.

Intervention results

- The intervention group had an average of 10 more days covered by effective contraceptive use during the 90 days following a first birth than the control group (52.6 vs. 62.2).
- By six months postpartum, 50% of the control group and 39% of the intervention group were using LARC.
- In analyses excluding LARC use, the intervention group had an average of 14 more days covered by effective contraceptive use 0–3 months postpartum (65.0 vs. 79.2) and 16 more covered days 4–6 months postpartum (39.2 vs. 54.9).

**Adaptable key components**<br>• Nurse home visiting program for pregnant women before 33 weeks gestation.			
**Level of gender responsiveness**<br>• Level 3 (Gender-sensitive). Focuses on decreasing women's gaps in use of effective methods after childbirth to reduce the incidence of unintended and short-interval pregnancies but does not address informed contraceptive decision-making nor how gender dynamics might be impacting women's experiences with the program.			
Furey, A., et al. (2019) (30)	Tanzania and South Africa	Women giving birth in 20 health facilities and receiving postpartum contraception services in Dar Es Salaam and Kilimanjaro regions in Tanzania (10 facilities) and the Western Cape region in South Africa (10 facilities) between 2015 and 2018 who were part of the UK Royal College of Obstetricians and Gynaecologists' Leading Safe Choices program. Conducted in collaboration with the Department of Health, Western Cape, South Africa and the Ministry of Health, Community Development, Gender, Elderly & Children in Tanzania. 526 health providers were trained (148 in Tanzania and 378 in South Africa). 60,503 women received PPFP.	Implementation research study with a pre-post design. An external evaluation was conducted to reflect on successes and challenges, and to aid in the development of future projects.
**Strategies that report success**<br>• The intervention was aimed to increase the uptake and choice of PPFP in Tanzania and South Africa through education and training of midwives, nurses, and non-specialist doctors on counseling and provision of PPFP, particularly PPIUD.<br>• In line with national policies, all contraceptive methods were provided free.<br>• The program used interactive classroom-based training to address negative attitudes, myths, and misconceptions, and to empower healthcare providers.<br>• The training was supplemented with on-the-job supervision and periodic refresher courses.<br>• The program also included a system of regular feedback using monitoring and evaluation data to improve uptake.<br>• A theory of change and detailed adaptive programmatic planning were used to achieve the program goals.			
**Intervention results**<br>• The proportion of women receiving immediate PPFP in the intervention facilities rose from 1% to 59% of all deliveries in Tanzania and from 82% to over 97% in South Africa.<br>• The PPIUD continuation rates at 6 months were high, with 95% in Tanzania and 88% in South Africa, although loss to follow up was 52%.<br>• Women's satisfaction was high, with 95% of them saying they would recommend PPIUD to other women.			
**Adaptable key components**<br>• Interactive classroom-based training addresses misconceptions and negative attitudes and empowers healthcare providers.<br>• Regular feedback using monitoring and evaluation data to improve uptake.<br>• On-the-job supervision and periodic refresher courses to aid knowledge and skills retention.<br>• Involvement of the Department or Ministry of Health from the outset for project ownership, partnership building, and sustainability.<br>• Use of a theory of change and detailed adaptive programmatic planning to achieve program goals.<br>• PPFP counseling training to support a woman's choice of contraception and for PPFP demand generation.			
**Results indicators**<br>• Continuation and satisfaction with method at 6 weeks and 6 months postpartum.			
**Level of gender responsiveness**<br>• Level 3 (Gender-sensitive). Focuses on improving the uptake and choice of postpartum FP but does not address informed contraceptive decision-making nor how gender dynamics might be impacting women's experiences with the program.			

Annex 2: Review and observational studies of strategies that report success in expanding post-pregnancy contraception

1. Review studies:

There were 17 review studies. Six were conducted in a single country and 11 in multiple countries. Combined, the studies were conducted in: Benin (1), Brazil (1), Burkina Faso (1), China (3), Congo (1), Democratic Republic of the Congo (1), Dominican Republic (1), Egypt (3), Ethiopia (3), Finland (1), Guinea (1), Kenya (2), Liberia (1), Madagascar (1), Malawi (2), Mexico (1), Mozambique (1), Nepal (2), Nigeria (1), South Africa (2), South Sudan (1), Spain (1), Sri Lanka (2), Tanzania (1), Turkey (1), Uganda (3), United Kingdom (3), United States (7), and Vietnam (1). All the studies were published in English. **Table A2a** shows the study characteristics of each publication by year, country, population studied, and methodological design for review studies.

Table A2a: Review studies identified in the scoping review by chronological order (2013-2023)

Reference and year	Country	Population (and sample size when applicable)	Methodological design
		**Reviews (N=17)**	
Hathaway, M., et al. (2014) (31)	United States	Not explicit	The methodological design of the paper is not explicitly mentioned.
Shah, I. H., et al. (2015) (32)	Multiple countries	Women seeking abortion care and those in the postpartum period.	Review paper on the importance of post-abortion contraception, emphasizing that women seeking abortions often have a strong desire to delay or limit childbearing and may face difficulties in obtaining contraception. Without prompt contraceptive advice and services, there is a clear risk of repeat abortion and potential harm to the woman's health and survival. The paper also introduces the topic of postpartum and post-abortion contraception, highlighting the efforts made to address unmet need for contraception during the postpartum period and the importance of contraceptive counseling and services following abortion. It presents key findings on postpartum contraceptive protection, use dynamics, method mix, and interventions to improve postpartum family planning
Blazer, C., et al. (2016) (33)	Sub-Saharan Africa, the Middle East and North Africa, and Asia	Women in the 12-month postpartum period in low- and middle-income countries.	Scoping review. Studies were considered if they focused on women from low- and middle-income countries within a year after giving birth. The interventions took place in both facility and community environments. Strategies were given to both women and men during antenatal care, labor, delivery, and postpartum. Only studies that gathered data on family planning during the postpartum period of the woman were included.
Huber, D., et al. (2016) (34)	Multiple countries spanning Asia, Africa, Latin America, or the post-Soviet states, Australia, Japan, Europe, or the United States	Not explicit	Review paper on postabortion care, postabortion contraception, incomplete abortion, abortion complications, dilation and curettage, misoprostol, and manual vacuum aspiration.
Lopez, L. M., et al. (2016) (35)	United States and China	Young people up to age 25.	Review of randomized controlled trials and non-randomized studies on educational interventions for improving contraceptive use among young people. Strategies had to be one to three sessions of 15 to 60 minutes each and may have been conducted with individuals or groups.
Harrison, M. S., et al. (2017) (36)	Sub-Saharan Africa, Southeast Asia, and Latin America	Women in the immediate postpartum	Review of literature on use of immediate postpartum long-acting reversible contraceptives in low- and middle-income countries.
Rees, H., et al. (2017) (37)	South Africa	Women of reproductive age.	Review of strategies that successfully promoted the provision of contraceptive implants and consideration for its use in South Africa.

Rogers, C., et al. (2017) (38)	Sri Lanka, Nepal, Vietnam, Mexico, Dominican Republic, Kenya, Mozambique, and Ethiopia	Women living in low-and middle-income countries who have undergone medical or surgical abortion.	The paper follows a systematic literature review methodology to document, analyze, and critique the accessibility of contraception and sexual and reproductive health information for women in low- and middle-income countries who have undergone medical or surgical abortion. The review utilized the PRISMA framework guidelines, flow diagram, and checklist to conduct the review. The Ovid (MEDLINE), ProQuest, Science Direct, Web of Science, PUBMED, and CINAHL databases were searched for relevant studies. Studies that met eligibility criteria were assessed for validity and analysis, and a narrative synthesis of characteristics and results of the included studies is presented.
Tappy, E., et al. (2017) (39)	United States	Postpartum women.	Not explicit
Brammeier, K., et al. (2018) (40)	United Kingdom		The methodological design of the paper is not explicitly mentioned in the provided sources.
Cwiak, C., et al. (2018) (41)	United States	Not explicit	Not explicit.
Wakuma, B., et al. (2020) (42)	Ethiopia	The study focused on postpartum women in Ethiopia. The systematic review and meta-analysis included 19 primary studies. The sample size in these studies ranged from 248 in Debre Berhan town to 1,109 in the Tigray region.	Systematic review and meta-analysis design, incorporating both published and unpublished studies. Data extraction was performed using Microsoft Excel, and analysis was conducted using STATA 14.1 version. The Joanna Briggs Institute Meta-Analysis of Statistics Assessment and Review Instrument (JBI-MAStARI) was used for quality appraisal. The PRISMA flow chart was followed to identify and select relevant studies. Subgroup analysis was computed to minimize underlying heterogeneity. The presence of publication bias was assessed by funnel plots and Egger's statistical tests.
Yemane, T. T., et al. (2021) (43)	Ethiopia, Malawi, Guinea, Madagascar, Benin, Liberia, Tanzania, Uganda, South Sudan, Democratic Republic of the Congo	Postpartum women in low-income countries of Sub-Saharan Africa. N=27,128.	Systematic review and meta-analysis of published and unpublished studies. The quality of the studies was evaluated using a modified version of the Newcastle-Ottawa Scale, with studies classified into low, medium, and high-quality categories. Data analysis was performed using STATA Version 14 software, with funnel plots, Egger's and Begg's tests used to assess publication bias. Heterogeneity was evaluated using Cochran's Q test and the I2 test, with a random effect model used in cases of heterogeneity.
Nelson, H. D., et al. (2022) (44)	United States, United Kingdom, and Finland	Women of reproductive age.	Systematic review and meta-analysis of randomized controlled trials (RCTs) evaluating the effectiveness of contraceptive counseling and provision interventions for women. The RCTs included in the review compared interventions providing contraceptive counseling, contraceptives, or both with usual care or an active control. The study used a research protocol developed in collaboration with methodological and content experts. The quality and applicability of each study were assessed by two investigators independently using criteria developed by the U.S. Preventive Services Task Force. The results were combined using a profile likelihood random-effects model. The strength of evidence and applicability for each key question and outcome were assessed based on the number, quality, and size of studies; the consistency of results between studies; and the directness of the evidence. Four RCTs focused on the immediate postpartum (all in the United States) and six on post-abortion (four in the US, three in the UK, and one in Finland).
Sothornwit, J., et al. (2022) (45)	United States, Uganda, Egypt, Brazil, and Sri Lanka	People who had just given birth in hospitals. The research included 16 studies with a total of 2,609 participants.	Systematic review and meta-analysis of randomized controlled trials (RCTs) of studies in which postpartum people were randomized individually. Data extraction included study design, methodology, population, outcomes, and related summary statistics. Statistical analyses were performed using RevMan 5, with a fixed-effect model used to combine data where studies were estimating the same underlying treatment effect. In cases of unexplained clinical heterogeneity or substantial statistical heterogeneity, a random-effects meta-analysis was used.
Hu, D., et al. (2023) (46)	Nepal, United Kingdom, China, South Africa, United States, China, Turkey, Nigeria, Egypt, Uganda, Burkina Faso, Kenya, Spain, Congo, Egypt, Malawi	Postpartum women aged 18 years or older, excluding those with HIV or with another condition. N=14,289.	Systematic review and meta-analysis, following the PRISMA guidelines. A systematic literature search was conducted in three databases until June 2022. The study included both randomized controlled trials and non-randomized trials. The study assessed the impact of service interventions on postpartum contraceptive use, prevention of repeat pregnancies, and induced abortions. The heterogeneity of the studies was assessed, and depending on the degree of heterogeneity, either a fixed or random effects model was applied.
Nabhan, A., et al. (2023) (47)	Multiple low-, middle-, and high-income countries	Post-pregnancy women and health providers.	Involves secondary data collection. The paper is a mixed-methods systematic review, combining quantitative, qualitative, and mixed method reports on scaling up and sustaining post pregnancy family planning. Electronic bibliographic databases were searched for relevant studies, and abstracts, titles, and full-text papers were assessed according to inclusion criteria. The Mixed Methods Appraisal Tool was used to assess the methodological quality of the included reports. A convergent integrated approach and deductive thematic synthesis were used to identify themes and sub-themes of strategies to scale up post pregnancy family planning. The health system building blocks were used to summarize barriers and facilitators. GRADE-CERQual was used to assess the confidence in the findings. The full review was reported according to the Preferred Reporting Items for Systematic Reviews and Meta-Analyses guidelines.

2. Observational studies:

There were 25 observational studies. Twenty-two observational studies were conducted in a single country and 3 in multiple countries. Combined, the studies were conducted in: Bangladesh (2), Democratic Republic of the Congo (1), Ethiopia (4), Ghana (2), India (2), Kenya (2), Mexico (1), Mozambique (1), Nepal (1), Nigeria (4), Pakistan (1), Rwanda (1), Senegal (1), Somalia (1), South Africa (1), Syria (1), Tanzania (1), Uganda (1), United Kingdom (2), United States (6), Yemen (1), and Zambia (1). All the studies were published in English. **Table A2b** shows the study characteristics of each publication by year, country, population studied, and methodological design for observational studies.

Table A2b: Observational studies identified in the scoping review by chronological order (2013-2023)

Reference and year	Country	Population (and sample size when applicable)	Methodological design
		**Observational (N=25)**	
Speizer, I. S., J. C, et al. (2013) (48)	Senegal	Women aged 15-49 from six cities (Dakar, Guédiawaye, Pikine, Mbao, Mbour, and Kaolack) within two years postpartum who participated through household surveys (n = 1,879) or through exit interviews after visiting high-volume health facilities. N=794.	Cross-sectional survey design, collecting data from six cities in Senegal. Data were collected from a representative household sample of women aged 15-49 within two years postpartum. Additional data were collected through exit interviews with women visiting high-volume health facilities for various services, including delivery, post-abortion care, postnatal care, and child immunization services. Descriptive analyses were used to examine women's exposure to family planning services. Logistic regression models were used to estimate the effect of integrated services on postpartum family planning use. The study adjusted for variables such as age, education, religion, type of union, wealth group, number of living children, and study site. The study protocol and informed consent process received ethical approval.
Mengesha, Z. B., et al. (2015) (49)	Ethiopia	Women aged 15 to 49 years (n=899) who delivered a child in the Dabat district of Northwest Ethiopia.	The study employed a house-to-house survey method to interview the participants. The sample included all women aged 15 to 49 years who delivered a baby between January 1, 2012, and December 31, 2012, in the Dabat district. The study protocol was reviewed and approved by the Institutional Review Board of the College of Medicine and Health Sciences of the University of Gondar. Consent was obtained from all participants to ensure ethical considerations.
Tisha, S., et al. (2015) (50)	Bangladesh	Ever-married women of reproductive ages 12-49 who had a live birth within five years before the survey. The sample size was 17,842 women selected from the 2011 Bangladesh Demographic and Health Survey data set.	The study used a two-stage stratified sample of households from the 2011 Bangladesh Demographic and Health Survey (BDHS) data set. The primary sampling unit for the survey was an Enumeration Area (EA), with 600 EAs selected based on probability proportional to the EA size. In the second stage, a systematic sample of 30 households on average was selected per EA. The Woman's Questionnaire was used to collect information from ever-married women aged 12-49 years. Descriptive analyses covering Pearson's chi-square test and a binary logistic regression model were applied to analyze the comparative contribution of various maternity and socio-demographic conjecturers of uptake of modern contraceptives during the postpartum period.
Eluwa, G., et al. (2016) (51)	Nigeria	Women delivering in 11 private health care facilities in six southern Nigerian states. N=728.	The study used a cross-sectional design, collecting demographic and reproductive health data using a standardized user intake form. Data collected included educational attainment, parity, number of living children, and previous use of contraception. Data were extracted from the women's intake forms and entered into Microsoft Excel, then imported into STATA 13.1 for statistical analysis. Bivariate logistic regression analysis was used to test associations between demographic and reproductive health variables, and uptake of postpartum IUD. Health care providers were trained on postpartum IUD service provision and received routine supportive supervision and mentoring. Quality technical assurance exercises were conducted to ensure quality postpartum IUD services.
Heller, R., et al. (2016) (52)	United Kingdom	The study focused on women in the postpartum period in Edinburgh, Scotland. The study examined 1,175 women attending for abortion and 4,713 postpartum women for inter-pregnancy intervals. Additionally, 250 women were surveyed about their contraceptive intentions prior to discharge from maternity service.	Retrospective analysis of databases for a 6-month period to examine the proportion of women attending for abortion who had given birth in the preceding 12 months, and the proportion of women giving birth after an inter-pregnancy interval of 12 months or less. A survey was conducted on women prior to discharge from maternity service about their contraceptive intentions. The data from the questionnaires were coded and entered into Microsoft Excel, and statistical analysis was conducted using IBM Statistical Package for Social Sciences (SPSS) software. Groups were compared by Chi-squared test of significance.
Rajan, S., et al. (2016) (53)	India	Women aged 15-49 who had a birth between 2011 and 2014 in Uttar Pradesh, India. N=2,733.	The study used a cross-sectional design, analyzing data from a survey conducted among women who had a birth between 2011 and 2014 in Uttar Pradesh, India. The survey collected data on women's exposure to family planning counseling during the antenatal, intrapartum, and postpartum periods. The study also controlled for baseline values of social and demographic measures associated with contraceptive use. The researchers used weights to account for the complex sampling design and make the sample representative of the selected cities. The study accounted for the correlated errors caused by the clustering of women within primary sampling units.

Shelton, J. D., et al. (2016) (54)	Nigeria	Married and unmarried women of reproductive age in Nigeria.	The study uses a descriptive approach, analyzing the success of various family planning programs in Nigeria. It includes an examination of the acceptability and effectiveness of long-acting reversible contraceptives. The study also considers the role of community health extension workers (CHEWs) and private-sector approaches in contraceptive provision. It assesses the demand for modern contraception and the socioeconomic status of women. The study also evaluates knowledge about contraception and regional differences in contraceptive use. Finally, it identifies system support activities needed for successful family planning, including supply chain management, human resources, policy and advocacy, and monitoring and evaluation.
Sodje, J. D., et al. (2016) (55)	Nigeria	Women undergoing delivery at eight medical facilities in southern Nigeria. N=374.	The research was a prospective analytical cohort study conducted at eight medical facilities in southern Nigeria from June 2014, to May 2015. All patients who delivered at the study sites during the study period were considered for eligibility. Data recorded included patient demographic information, delivery rate, number of eligible patients counselled, number of patients who agreed to participate, actual number of patients who received PPIUDs, complication rates, scheduled follow-up visit attendance rates, patient satisfaction at 6-week follow-up, future PPIUD use intentions, and recommendations to others. Participants were scheduled to attend 14-day and 6-week follow-up post PPIUD insertion. Data were analyzed using SPSS version 20.0, expressed as absolute numbers and percentages, and compared using the kh2 or Fisher exact test.
Abraha, T. H., et al. (2017) (56)	Ethiopia	Women in the extended postpartum period in northern Ethiopia. N=610.	The study employed a community-based cross-sectional design to assess postpartum modern contraceptive use among women in northern Ethiopia. Data collection was conducted from March to April 2015, and analysis was performed using Epi Info version 7 and Stata version 12. The study used both bivariate and multivariate logistic regression models to identify the determinants of postpartum modern contraceptive use.
Jalang'o, R., et al. (2017) (57)	Kenya	Postpartum women who had brought their children for the second dose of measles vaccine between 18 and 24 months in a public referral hospital in rural Kenya. Mothers whose children were aged below 12 months and more than 24 months were excluded from the study. N=365.	The study employed a mixed method approach, incorporating both quantitative and qualitative methods of data collection. Quantitative data were collected through structured questionnaires, capturing socio-demographic characteristics, fertility, knowledge, use, and access to contraceptives. Chi square tests were used to determine the relationship between uptake of postpartum family planning and various factors. Qualitative data were collected through focus group discussions with mothers and in-depth interviews with service providers. The study also explored perceptions on family planning methods, use, availability, access, and barriers to uptake.
Morhe, E. S. K., et al. (2017) (58)	Ghana	Women who attended the well-baby clinic of Komfo Anokye Teaching Hospital, Kumasi, at 6–24 months after delivery. N=200.	The study employed a descriptive cross-sectional survey design. Participants were consecutively recruited and interviewed using semi-structured questionnaires. Data were collected on demographics, exposure to family planning counseling, message content, and postpartum contraceptive choices. The interviews were conducted face-to-face in private, using either English or a local language understood by the participant. Statistical tests such as the Pearson kh2 test, Fisher exact test, Student t test, and analysis of variance were used for data analysis.
Potter, J. E., et al. (2017) (59)	Mexico and United States	Mexican women in Mexico and Texas, United States, at six months postpartum.	The study employed a mixed-methods approach, combining quantitative and qualitative data collection techniques. A prospective study was conducted, with participants interviewed at 6 months postpartum. In-depth interviews were conducted with a subset of participants who had changed their mind about their ideal contraceptive method or had a consistent preference. The study also included a case study contrasting the provision of contraception in Texas and Mexico.
Achwoka, D., et al. (2018) (60)	Kenya	Women 8-10 months postpartum attending infant immunization visits at maternal and child health clinics in Kenya. N=955.	The research employed a national, cross-sectional study design. The study included postpartum women attending infant immunization visits at 120 Kenyan maternal and child health clinics. The analysis focused on women who resumed sexual activity postpartum and did not desire a child within 2 years, classifying them as having a need for family planning. Modern contraception methods were defined, and statistical analysis was conducted using univariate Poisson generalized linear models with a log-link function. Survey weights and clinic-level clustering adjustments were applied to account for the sampling design and ensure representativeness.
Benson, J., et al. (2018) (61)	Ethiopia, Ghana, Nigeria, South Africa, Zambia, Bangladesh, Nepal, India.	The study population consisted of women seeking abortion services in public-sector health facilities across eight countries in Africa and Asia. The sample size was 319,385 women: 169,791 women from Africa and 149,594 women from Asia.	The study is a cross-sectional analysis of service delivery monitoring data collected from public-sector health facilities in eight countries. Data were collected through a standardized monitoring form administered at each site at the start of the intervention. The study examined factors contributing to differences in contraceptive uptake and developed recommendations for improving postabortion contraceptive services. The study is not an impact evaluation and did not compare pre- and post-intervention results due to poor data collection prior to interventions.
Croan, L., et al. (2018) (62)	United Kingdom	Women discharged from hospital after birth who lived in South East Edinburgh, Scotland. N=40.	Questionnaire administered to women who had received a postpartum subdermal implant at home immediately after insertion. The authors discuss the role of community midwives in providing postnatal contraception at home to new mothers using a topical anesthetic spray.

Cross-Barnet, C., et al. (2018) (63)	United States	Postpartum women enrolled in the Strong Start for Mothers and Newborns Initiative, a program for Medicaid beneficiaries. N=10,374 for the survey, N=629 for interviews with staff, and 122 focus groups with 887 women.	The paper utilized a mixed-methods approach, combining qualitative and quantitative data collection and analysis methods. Individual-level data were collected through participant survey instruments and a medical chart review. Qualitative case studies were conducted annually for the first 3 years of the program, including interviews with staff and focus groups with Strong Start participants. The data were cleaned, organized by theme, and coded using the software program NVivo. Multiple rounds of qualitative database testing were conducted to ensure high intercoder and intracoder reliability.
DeSisto, C. L., et al. (2018) (64)	United States	Women with a live birth paid for by Georgia Medicaid who received an immediate postpartum long-acting reversible contraception. N=51.	The study employed a pilot design, using a novel population-based sampling technique in partnership with Georgia Department of Community Health. The researchers sampled all women with a live birth paid for by Georgia Medicaid during November 2015 through February 2017 who received an immediate postpartum LARC. A one-to-one match was used to sample women who did not receive immediate postpartum LARC. Women were contacted via telephone for a 25-30 min interview regarding their knowledge, attitudes, and behaviors related to immediate postpartum LARC and their satisfaction with postpartum contraception. Descriptive statistics and components of informed choice were calculated overall and by receipt of immediate postpartum LARC, using chi-square tests to calculate differences by group.
Zimmerman, L. A., et al. (2019) (65)	Ethiopia	Postpartum women in the Southern Nations Nationalities and Peoples Region of Ethiopia. Initially, 329 women who were 6 or more months pregnant met the inclusion criteria from a sample of 10,399 households. The final analysis included 307 women who completed the survey at 6 months postpartum.	The study used a longitudinal design aimed to establish whether PPFP counseling was being provided in ANC and postnatal care services and whether it improved the uptake of PPFP. Data were collected through interviews at screening (at least 6 months of pregnancy), and 7-days, 6-weeks, and 6-months postpartum using smartphones programmed with Open Data Kit. The study used weighted parametric survival analysis with Weibull distribution to assess the effect of postpartum counseling on contraceptive uptake, after adjusting for intention to use FP, wantedness of the index pregnancy, delivery location, amenorrhea, exclusive breastfeeding, residence, parity, and education.
Faini, D., et al. (2020) (66)	Tanzania	Female sex workers (FSWs) in Dar es Salaam, Tanzania. The sample size was 11 FSWs who were part of a HIV vaccine preparedness cohort and had a positive pregnancy test.	The study employed a qualitative research design using in-depth interviews. Grounded theory approach was used to analyze the interview transcripts. Open and selective coding was performed using Nvivo software. Theoretical coding was performed to explain how the codes linked with each other in a particular category and between categories. A model grounded in the data was constructed to illustrate how the codes, the categories, and the core-category related with each other and with respect to the study research question. The interview guide evolved with subsequent interviews accommodating emerging themes based on responses provided by preceding interviews.
Tusubira, A. K., et al. (2020) (67)	Uganda	Women with HIV attending postpartum care at six health facilities in Kabarole district, Uganda. N=369.	The study employed a cross-sectional design, conducted between April and May 2016. Participants were selected using systematic sampling from health facilities stratified by level. Data were collected using structured questionnaires administered to women who had delivered within the last two to 18 months. The study estimated a modified Poisson regression model to examine variations in the use of modern contraceptive methods by various characteristics of participants. Unmet need for contraception was estimated using the revised algorithm for measuring such need from demographic and health surveys data.
Brant, A. R., et al. (2021) (68)	United States	All patients giving birth at three Cleveland Clinic Ohio hospitals from July 2015 to June 2019. The sample size included 17,848 deliveries before the policy implementation and 18,555 deliveries after the policy implementation.	The study employed a retrospective chart review methodology to assess the rate of immediate postpartum LARC use before and after the implementation of a state policy mandated inpatient access to LARC after delivery and before discharge. The researchers compared all patients who received inpatient postpartum LARC to a 1:3 matched sample of patients who did not receive LARC, matched by delivery date and location. The electronic medical record was reviewed to identify new pregnancies occurring within 12 months post-delivery. The study also described the characteristics of patients who use immediate postpartum period LARC when it is universally available and compared pregnancy rates within 12 months postpartum among immediate postpartum period LARC users and nonusers.
Gallagher, M. C., et al. (2021) (69)	Democratic Republic of the Congo, Somalia, Rwanda, Yemen, Syria, and Pakistan.	Postpartum women from complex humanitarian settings in six countries. Catchment population is 2.5 million. Retrospective service delivery data from July 2016 to December 2019 were analyzed.	The study used a comparative design, examining trends in IPP LARC uptake across six countries with different intervention intensities. The study compared countries with higher-intensity IPPFP interventions to those providing standard care. Tests of association were performed to assess the significance of differences between higher-IPPFP intervention countries and standard-care countries. An analysis of variance was used to compare the proportion of IPP LARC uptake between higher-intensity intervention programs and standard care programs.
Koch, S. K., et al. (2022) (70)	United States	Patients who delivered at a large, urban, tertiary medical center in Missouri, one year before and after a change in Medicaid coverage in October 2016 that introduced a reimbursement program for immediate postpartum LARC. N=6,233.	Retrospective cohort design, comparing patients who delivered at a large, urban, tertiary medical center one year before and after a change in Medicaid coverage. Patients were identified through the electronic medical record and excluded if they delivered prior to 24 weeks gestation or had a contraindication to immediate postpartum LARC. The primary outcome was the placement of immediate postpartum LARC, which was examined overall and stratified by insurance type. Multivariable logistic regression was used to determine the impact of the policy change while adjusting for appropriate confounders.

Lagon, E. P., et al. (2023) (71)	United States	Patients who delivered in a teaching hospital in Massachusetts during the pre-COVID-19 period (2019) and the COVID-19 period (2020). N=1,672.	Retrospective cohort study. The study period was the first six months of the COVID-19 pandemic (March 2020 to September 2020) compared to the same period in the previous year (March 2019 to September 2019). Data were collected from hospital records and anonymized. Statistical analyses were performed using STATA, including Student's t test, Wilcoxon rank-sum test, Chi-square tests, and multivariable logistic regression. The primary exposure was the time period of delivery: before versus during the COVID-19 pandemic. Primary outcomes were contraception and sterilization rates at 12 weeks postpartum as well as postpartum visit attendance.
Sack, D. E., et al. (2023) (72)	Mozambique	Sero-concordant partners with HIV in rural Mozambique. The sample size included 38 recently pregnant people and 26 of their partners, totaling 64 participants.	Qualitative component of the *Homens para Saúde Mais* (HoPS) trial, which included in-depth interviews with participants. Trained personnel conducted interviews in the participants' preferred language, with responses translated into Portuguese and then English. Participants were randomly selected from six of the 12 intervention sites of the HoPS trial. The authors used MAXQDA2020(r) software for coding and analysis, generating 14 deductive codes and 3 inductive codes across three themes.

Annex 3: Documentation of results indicators used to assess the success of strategies from a gender perspective, focusing on indicators relevant to measurements of bodily autonomy.

Error! Reference source not found.**A3** shows a list of indicators based on the strategies to measure and evaluate the rights-based counseling and provision of post-pregnancy contraception. They are structured around three themes: counseling and education, service delivery and quality of care, and follow-up.

Table A3: Results indicators to measure and evaluate the rights-based counseling and provision of post-pregnancy contraception

**A.**	**Counseling and Education**	
1.	Women reporting receiving counseling for post-pregnancy contraception during antenatal care.	
2.	Women reporting receiving counseling for post-pregnancy contraception after admission to the hospital for delivery.	
3.	Women given the opportunity to ask questions during counseling.	
4.	Women who recalled benefits and/or disadvantages of post-pregnancy contraception that trainers were expected to mention during counseling.	
5.	Women counseled who gave consent to receive post-pregnancy contraception.	
6.	Providers' ability to appropriately perform key contraceptive indicators:	
	a.	Offer comprehensive contraceptive counseling (including opportunities for method removal if desired).
	b.	Offer recommendations for the timely initiation of a method after abortion.
	c.	Assess the woman's SRH intentions (including desire for future pregnancy).
	d.	Provide method information and supply.
	e.	Document services on the woman's contraception card.
7.	Satisfaction with counseling (very satisfied, satisfied, somewhat satisfied, not very satisfied, not at all satisfied).	
8.	Likability and satisfaction of counseling:	
	a.	Liked the intervention (a lot, a little, not much, not at all).
	b.	What was liked (missed postpartum follow up, not planning on attending postpartum follow up, in need of birth control, or convenient, other, none).
	c.	What was not liked (no time to stay longer, too tired after baby's visit, uncomfortable seeing new provider, children were there, worried about insurance, other, none).
	d.	Chose to use these services again (definitely, probably, probably not, definitely not).
	e.	Reasons for declining the intervention: (no time to stay longer, too tired to stay longer, uncomfortable seeing new provider, children were present, worried about insurance coverage, do not want birth control, already have birth control, already have a scheduled postpartum visit, other, none).
	f.	Satisfaction with counseling (very satisfied, somewhat satisfied, not very satisfied, not at all satisfied).
	g.	Comfortable with birth control at this visit: very comfortable, somewhat comfortable, somewhat uncomfortable, very uncomfortable).
	h.	Convenience of visit (very convenient, somewhat convenient, neither convenient nor inconvenient, somewhat inconvenient, very inconvenient).
	i.	Time spent on counseling (too little time, just the right amount of time, a little too much time, way too much time).
	j.	Received new birth control method today (yes, no).
	k.	Recommend to a friend (definitely, probably, probably not, definitely not).
	l.	Rating of quality of care (excellent, very good, good, fair, poor).
**B.**	**Service Delivery and Quality of Care**	
9.	Time from delivery or abortion to uptake of modern contraception.	
10.	Women who received their preferred post-pregnancy method before discharge.	
11.	Post-pregnancy LARC users' anxiety Likert scale (1 = no anxiety or pain to 10 = maximum anxiety or pain).	
12.	Comfort with implant or IUD insertion at the visit (very comfortable, somewhat comfortable, somewhat uncomfortable, very uncomfortable).	
13.	Satisfaction with the method chosen (very satisfied, satisfied, somewhat satisfied, not very satisfied, not at all satisfied).	
14.	PPIUD expulsion rate as a measure of quality of care.	
15.	Referrals for STI treatment.	
**C.**	**Follow-Up**	
16.	Continuation and satisfaction with the method at 6 weeks and 6 months postpartum.	
17.	Women who desire to get pregnant again who have their LARC method removed.	

Note: Prepared by the authors based on multiple sources (1, 4, 5, 8, 10, 12, 15, 17-19, 22-26, 28, 30).

References

1. Tawfik Y, Rahimzai M, Ahmadzai M, Clark PA, Kamgang E. Integrating family planning into postpartum care through modern quality improvement: experience from Afghanistan. Global Health: Science and Practice. 2014;2(2):226-33.
2. Curry DW, Rattan J, Nzau JJ, Giri K. Delivering high-quality family planning services in crisis-affected settings I: program implementation. Glob Health Sci Pract. 2015;3(1):14-24.
3. Samuel M, Fetters T, Desta D. Strengthening Postabortion Family Planning Services in Ethiopia: Expanding Contraceptive Choice and Improving Access to Long-Acting Reversible Contraception. Global Health: Science and Practice. 2016;4(S2):S60-S72.
4. Mbehero F, Momanyi R, Hesel K. Facilitating Uptake of Post-abortion Contraception for Young People in Kenya. Front Glob Womens Health. 2021;2:733957.
5. Adanikin AI, Onwudiegwu U, Loto OM. Influence of multiple antenatal counselling sessions on modern contraceptive uptake in Nigeria. Eur J Contracept Reprod Health Care. 2013;18(5):381-7.
6. Cooper CM, Ahmed S, Winch PJ, Pfitzer A, McKaig C, Baqui AH. Findings from the use of a narrative story and leaflet to influence shifts along the behavior change continuum toward postpartum contraceptive uptake in Sylhet District, Bangladesh. Patient Educ Couns. 2014;97(3):376-82.
7. Huang Y, Merkatz R, Zhu H, Roberts K, Sitruk-Ware R, Cheng L, et al. The free perinatal/postpartum contraceptive services project for migrant women in Shanghai: effects on the incidence of unintended pregnancy. Contraception. 2014;89(6):521-7.
8. Pleah T, Hyjazi Y, Austin S, Diallo A, Dao B, Waxman R, et al. Increasing Use of Postpartum Family Planning and the Postpartum IUD: Early Experiences in West and Central Africa. Glob Health Sci Pract. 2016;4 Suppl 2(Suppl 2):S140-52.
9. Chukwumalu K, Gallagher MC, Baunach S, Cannon A. Uptake of postabortion care services and acceptance of postabortion contraception in Puntland, Somalia. Reprod Health Matters. 2017;25(51):48-57.
10. Gbagbo FY, Elzohry AAM. Post Abortion Contraception Model: A Comprehensive Package for Improving Safe Abortion Care in Developing Countries. Journal of Family Medicine. 2018;1(1):12-21.
11. Ingabire R, Nyombayire J, Hoagland A, Da Costa V, Mazzei A, Haddad L, et al. Evaluation of a multi-level intervention to improve postpartum intrauterine device services in Rwanda. Gates Open Res. 2018;2:38.
12. Lori JR, Chuey M, Munro-Kramer ML, Ofosu-Darkwah H, Adanu RMK. Increasing postpartum family planning uptake through group antenatal care: a longitudinal prospective cohort design. Reprod Health. 2018;15(1):208.
13. Torres LN, Turok DK, Clark EAS, Sanders JN, Godfrey EM. Increasing IUD and Implant Use Among Those at Risk of a Subsequent Preterm Birth: A Randomized Controlled Trial of Postpartum Contraceptive Counseling. Womens Health Issues. 2018;28(5):393-400.
14. Wendot S, Scott RH, Nafula I, Theuri I, Ikiugu E, Footman K. Evaluating the impact of a quality management intervention on post-abortion contraceptive uptake in private sector clinics in western Kenya: a pre- and post-intervention study. Reprod Health. 2018;15(1):10.
15. Karra M, Pearson E, Pradhan E, de Silva R, Samarasekera A, Canning D, et al. The effect of a postpartum IUD intervention on counseling and choice: Evidence from a cluster-randomized stepped-wedge trial in Sri Lanka. Trials. 2019;20(1):407.
16. Mossie MY, Pfitzer A, Yusuf Y, Wondimu C, Bazant E, Bansal V, et al. Counseling at all contacts for postpartum contraceptive use: can paper-based tools help community health workers improve continuity of care? A qualitative study from Ethiopia. Gates Open Res. 2019;3:1652.
17. Pradhan E, Canning D, Shah IH, Puri M, Pearson E, Thapa K, et al. Integrating postpartum contraceptive counseling and IUD insertion services into maternity care in Nepal: results from stepped-wedge randomized controlled trial. Reprod Health. 2019;16(1):69.

18. Stephens B, Mwandalima IJ, Samma A, Lyatuu J, Mimno K, Komwihangiro J. Reducing Barriers to Postabortion Contraception: The Role of Expanding Coverage of Postabortion Care in Dar es Salaam, Tanzania. Glob Health Sci Pract. 2019;7(Suppl 2):S258-s70.
19. Haider S, Stoffel C, Rankin K, Uesugi K, Handler A, Caskey R. A Novel Approach to Postpartum Contraception Provision Combined with Infant Care: A Randomized, Controlled Trial. Womens Health Issues. 2020;30(2):83-92.
20. Huber-Krum S, Khadka A, Pradhan E, Rohr J, Puri M, Maharjan D, et al. The effect of antenatal counseling and intrauterine device insertion services on postpartum contraceptive use in Nepal: Results from a stepped-wedge randomized controlled trial. Contraception. 2020;101(6):384-92.
21. Lacy MM, McMurtry Baird S, Scott TA, Barker B, Zite NB. Statewide quality improvement initiative to implement immediate postpartum long-acting reversible contraception. Am J Obstet Gynecol. 2020;222(4S):S910 e1-S e8.
22. Pearson E, Senderowicz L, Pradhan E, Francis J, Muganyizi P, Shah I, et al. Effect of a postpartum family planning intervention on postpartum intrauterine device counseling and choice: evidence from a cluster-randomized trial in Tanzania. BMC Womens Health. 2020;20(1):102.
23. Reyes-Lacalle A, Montero-Pons L, Manresa-Dominguez JM, Cabedo-Ferreiro R, Seguranyes G, Falguera-Puig G. Perinatal contraceptive counselling: Effectiveness of a reinforcement intervention on top of standard clinical practice. Midwifery. 2020;83:102631.
24. Sitrin D, Jima GH, Pfitzer A, Wondimu C, Belete TW, Pleah T, et al. Effect of integrating postpartum family planning into the health extension program in Ethiopia on postpartum adoption of modern contraception. Journal of Global Health Reports. 2020;4.
25. Wu WJ, Tiwari A, Choudhury N, Basnett I, Bhatt R, Citrin D, et al. Community-based postpartum contraceptive counselling in rural Nepal: a mixed-methods evaluation. Sex Reprod Health Matters. 2020;28(2):1765646.
26. Espey J, Ingabire R, Nyombayire J, Hoagland A, Da Costa V, Mazzei A, et al. Postpartum long-acting contraception uptake and service delivery outcomes after a multilevel intervention in Kigali, Rwanda. BMJ Sex Reprod Health. 2021;47(3):173-8.
27. Karra M, Maggio D, Guo M, Ngwira B, Canning D. The causal effect of a family planning intervention on women's contraceptive use and birth spacing. Proc Natl Acad Sci U S A. 2022;119(22):e2200279119.
28. Ijarotimi O, Ijarotimi I, Ubom A, Sowemimo O, Orji E. The effect of integration of family planning education with infant vaccination visits on the uptake of postpartum family planning in Ile-Ife, Nigeria. J Obstet Gynaecol. 2023;43(1):2186774.
29. Melnick AL, Rdesinski RE, Marino M, Jacob-Files E, Gipson T, Kuyl M, et al. Randomized Controlled Trial of Home-Based Hormonal Contraceptive Dispensing for Women At Risk of Unintended Pregnancy. Perspect Sex Reprod Health. 2016;48(2):93-9.
30. Furey A, Fiander A. Postpartum contraception - the experience and findings from the Leading Safe Choices programme in Tanzania and South Africa. BJOG. 2019;126(11):1301-4.
31. Hathaway M, Torres L, Vollett-Krech J, Wohltjen H. Increasing LARC utilization: any woman, any place, any time. Clin Obstet Gynecol. 2014;57(4):718-30.
32. Shah IH, Santhya KG, Cleland J. Postpartum and Post-Abortion Contraception: From Research to Programs. Stud Fam Plann. 2015;46(4):343-53.
33. Blazer C, Prata N. Postpartum family planning: current evidence on successful interventions. Open Access J Contracept. 2016;7:53-67.
34. Huber D, Curtis C, Irani L, Pappa S, Arringtone L. Postabortion Care: 20 Years of Strong Evidence on Emergency Treatment, Family Planning, and Other Programming Components. Global Health: Science and Practice. 2016;4(3):481-94.
35. Lopez LM, Grey TW, Tolley EE, Chen M. Brief educational strategies for improving contraception use in young people. Cochrane Database Syst Rev. 2016;3(3):CD012025.
36. Harrison MS, Goldenberg RL. Immediate postpartum use of long-acting reversible contraceptives in low- and middle-income countries. Matern Health Neonatol Perinatol. 2017;3:24.

37. Rees H, Pillay D, Mullick S, Chersich M. Strengthening implant provision and acceptance in South Africa with the 'Any woman, any place, any time' approach: An essential step towards reducing unintended pregnancies. S Afr Med J. 2017;107(11):939-44.
38. Rogers C, Dantas JAR. Access to contraception and sexual and reproductive health information post-abortion: a systematic review of literature from low- and middle-income countries. J Fam Plann Reprod Health Care. 2017;43(4):309-18.
39. Tappy E, Jamshidi R. Postpartum LARC: Best Practices, Policy and Public Health Implications. Current Obstetrics and Gynecology Reports. 2017;6(4):310-7.
40. Brammeier K, Cutter J, Cook S, Scherf C. The Cardiff postpartum family planning initiative: improving provision of postpartum contraception. BMJ Sex Reprod Health. 2018.
41. Cwiak C, Cordes S. Postpartum intrauterine device placement: a patient-friendly option. Contracept Reprod Med. 2018;3:3.
42. Wakuma B, Mosisa G, Etafa W, Mulisa D, Tolossa T, Fetensa G, et al. Postpartum modern contraception utilization and its determinants in Ethiopia: A systematic review and meta-analysis. PLoS One. 2020;15(12):e0243776.
43. Yemane TT, Bogale GG, Egata G, Tefera TK. Postpartum Family Planning Use and Its Determinants among Women of the Reproductive Age Group in Low-Income Countries of Sub-Saharan Africa: A Systematic Review and Meta-Analysis. Int J Reprod Med. 2021;2021:5580490.
44. Nelson HD, Cantor A, Jungbauer RM, Eden KB, Darney B, Ahrens K, et al. Effectiveness and Harms of Contraceptive Counseling and Provision Interventions for Women : A Systematic Review and Meta-analysis. Ann Intern Med. 2022;175(7):980-93.
45. Sothornwit J, Kaewrudee S, Lumbiganon P, Pattanittum P, Averbach SH. Immediate versus delayed postpartum insertion of contraceptive implant and IUD for contraception. Cochrane Database Syst Rev. 2022;10(10):CD011913.
46. Hu D, Tang Y, Pei K. Strategies for Improving Postpartum Contraception Compared With Routine Maternal Care: A Systematic Review and Meta-Analysis. Int J Public Health. 2023;68:1605564.
47. Nabhan A, Kabra R, Allam N, Ibrahim E, Abd-Elmonem N, Wagih N, et al. Implementation strategies, facilitators, and barriers to scaling up and sustaining post pregnancy family planning, a mixed-methods systematic review. BMC Womens Health. 2023;23(1):379.
48. Speizer IS, Fotso JC, Okigbo C, Faye CM, Seck C. Influence of integrated services on postpartum family planning use: a cross-sectional survey from urban Senegal. BMC Public Health 2013;13(752):9.
49. Mengesha ZB, Worku AG, Feleke SA. Contraceptive adoption in the extended postpartum period is low in Northwest Ethiopia. BMC Pregnancy Childbirth. 2015;15:160.
50. Tisha S, Haque SMR, Tabassum M. Antenatal Care, an Expediter for Postpartum Modern Contraceptive Use. Research in Obstetrics and Gynecology. 2015;3(2):22-31.
51. Eluwa G, Atamewalen R, Odogwu K, Ahonsi B. Success Providing Postpartum Intrauterine Devices in Private-Sector Health Care Facilities in Nigeria: Factors Associated With Uptake. Glob Health Sci Pract. 2016;4(2):276-83.
52. Heller R, Cameron S, Briggs R, Forson N, Glasier A. Postpartum contraception: a missed opportunity to prevent unintended pregnancy and short inter-pregnancy intervals. J Fam Plann Reprod Health Care. 2016;42(2):93-8.
53. Rajan S, Speizer IS, Calhoun LM, Nanda P. Counseling during Maternal and Infant Health Visits and Postpartum Contraceptive use in Uttar Pradesh, India. Int Perspect Sex Reprod Health. 2016;42(4):167-78.
54. Shelton JD, Finkle C. Leading With LARCs in Nigeria: The Stars Are Aligned to Expand Effective Family Planning Services Decisively. Global Health: Science and Practice. 2016;4(2):179-85.
55. Sodje JD, Enaruna NO, Ehigiegba AE, Aromeh CO, Atamewalen M. Feasibility, acceptability, and uptake of postpartum intrauterine contraceptive devices in southern Nigeria. Int J Gynaecol Obstet. 2016;135(2):149-53.

56. Abraha TH, Teferra AS, Gelagay AA. Postpartum modern contraceptive use in northern Ethiopia: prevalence and associated factors. Epidemiol Health. 2017;39:e2017012.
57. Jalang'o R, Thuita F, Barasa SO, Njoroge P. Determinants of contraceptive use among postpartum women in a county hospital in rural KENYA. BMC Public Health. 2017;17(1):604.
58. Morhe ESK, Ankobea F, Asubonteng GO, Opoku B, Turpin CA, Dalton VK. Postpartum contraceptive choices among women attending a well-baby clinic in Ghana. Int J Gynaecol Obstet. 2017;138(2):219-24.
59. Potter JE, Hubert C, White K. The Availability and Use of Postpartum LARC in Mexico and Among Hispanics in the United States. Matern Child Health J. 2017;21(9):1744-52.
60. Achwoka D, Pintye J, McGrath CJ, Kinuthia J, Unger JA, Obudho N, et al. Uptake and correlates of contraception among postpartum women in Kenya: results from a national cross-sectional survey. Contraception. 2018;97(3):227-35.
61. Benson J, Andersen K, Brahmi D, Healy J, Mark A, Ajode A, et al. What contraception do women use after abortion? An analysis of 319,385 cases from eight countries. Glob Public Health. 2018;13(1):35-50.
62. Croan L, Craig A, Scott L, Cameron ST, Lakha F. Increasing access to contraceptive implants in the postnatal period via a home insertion service by community midwives. BMJ Sex Reprod Health. 2018;44(1):61-4.
63. Cross-Barnet C, Courtot B, Hill I, Benatar S, Cheeks M, Markell J. Facilitators and Barriers to Healthy Pregnancy Spacing among Medicaid Beneficiaries: Findings from the National Strong Start Initiative. Womens Health Issues. 2018;28(2):152-7.
64. DeSisto CL, Handler A, Haider S, Caskey R, Peacock N, Kottke M, et al. Women's informed choice and satisfaction with immediate postpartum long-acting reversible contraception in Georgia. Contracept Reprod Med. 2018;3:19.
65. Zimmerman LA, Yi Y, Yihdego M, Abrha S, Shiferaw S, Seme A, et al. Effect of integrating maternal health services and family planning services on postpartum family planning behavior in Ethiopia: results from a longitudinal survey. BMC Public Health. 2019;19(1):1448.
66. Faini D, Munseri P, Bakari M, Sandström E, Faxelid E, Hanson C. "I did not plan to have a baby. This is the outcome of our work": a qualitative study exploring unintended pregnancy among female sex workers. BMC Womens Health. 2020;20(1):267.
67. Tusubira AK, Kibira SPS, Makumbi FE. Modern contraceptive use among postpartum women living with HIV attending mother baby care points in Kabarole District, Uganda. BMC Womens Health. 2020;20(1):78.
68. Brant AR, Kollikonda S, Yao M, Mei L, Emery J. Use of Immediate Postpartum Long-Acting Reversible Contraception Before and After a State Policy Mandated Inpatient Access. Obstet Gynecol. 2021;138(5):732-7.
69. Gallagher MC, Morris CN, Fatima A, Daniel RW, Shire AH, Sangwa BMM. Immediate Postpartum Long-Acting Reversible Contraception: A Comparison Across Six Humanitarian Country Contexts. Front Glob Womens Health. 2021;2:613338.
70. Koch SK, Paul R, Addante AN, Brubaker A, Kelly JC, Raghuraman N, et al. Medicaid reimbursement program for immediate postpartum long-acting reversible contraception improves uptake regardless of insurance status. Contraception. 2022;113:57-61.
71. Lagon EP, Mauney L, Onwuzurike C, Shahawy S, Schaefer K, Starosta A, et al. An assessment of postpartum contraception rates with evolving care during the COVID-19 pandemic. Sex Reprod Healthc. 2023;36:100844.
72. Sack DE, Emílio A, Graves E, Matino A, Paulo P, Aboobacar AU, et al. Attitudes and perceptions towards postpartum contraceptive use among seroconcordant partners with HIV in rural Mozambique: a qualitative study. Glob Health Res Policy. 2023;8(1):7.
